# Thromboembolic events in people with cancer during the COVID-19 pandemic: case-control study*

**DOI:** 10.1590/1518-8345.7075.4266

**Published:** 2024-08-19

**Authors:** Vivian Cristina Gama Souza Lima, Paulo Jorge Pereira Alves, Patrícia dos Santos Claro Fuly

**Affiliations:** 1 Universidade Federal Fluminense, Escola de Enfermagem Aurora de Afonso Costa, Niterói, RJ, Brazil.; 2 Ministério da Saúde, Instituto Nacional do Câncer, Rio de Janeiro, RJ, Brazil.; 3 Universidade Católica Portuguesa, Porto, Portugal.

**Keywords:** Oncology Nursing, Oncology, COVID-19, Thrombosis, Neoplasms, Case-Control Studies

## Abstract

**Objective:**

to analyze the association between coronavirus disease infection and thromboembolic events in people with cancer in the first year of the pandemic.

**Method:**

case-control study carried out by collecting medical records. The selected cases were adults with cancer, diagnosed with a thromboembolic event, treated in the selected service units during the first year of the pandemic. The control group included adults with cancer without a diagnosis of a thromboembolic event. Pearson’s chi-square test was applied to verify the association between risk factors and the outcome and logistic regression techniques were applied to identify the odds ratio for the occurrence of a thromboembolic event.

**Results:**

there were 388 cases and 440 control cases included in the study (ratio 1/1). Females predominated, who were white, with mean age of 58.2 (±14.8) years. Antineoplastic chemotherapy was the most used treatment and coronavirus disease was identified in 11.59% of participants. In the case group, deep vein thrombosis was more prevalent.

**Conclusion:**

the study confirmed the hypothesis that coronavirus disease infection did not increase the chance of thromboembolic events in people with cancer. For the population studied, the factors that were associated with these events were those related to cancer and its treatment.

## Highlights

**(1)** Deep vein thrombosis was what prevailed in the studied population.

**(2)** Chemotherapy increased the chance of thromboembolic events by 65%.

**(3)** Thromboembolic events showed a significant association with a higher death rate as the outcome.

**(4)** COVID-19 did not increase the risk of thromboembolic events in people with cancer.

## Introduction

Cancer is one of the main public health problems in Brazil and the world. It encompasses more than 100 types of diseases, and together with cardiovascular diseases, respiratory diseases and Diabetes Mellitus, it makes up the group of Chronic Non-Communicable Diseases (NCDs), responsible for around 70% of all deaths in the world^1^ .

The increasing incidence of oncological diseases and the complexity of caring for this individual involves several aspects, including the risk of developing venous thromboembolism (VTE). It is known that there is a close relationship between oncological disease and VTE, with an increased risk of these people developing a thromboembolic event when compared to the general population. A cohort study carried out in Spain compared the incidence rates of thromboembolism in populations with and without cancer in the years between 1997 and 2017. The cumulative incidence of VTE 12 months after cancer diagnosis was 2.3% in the cancer and 0.35% in the non-cancer cohort. Furthermore, the 12-month incidence in the cancer cohort increased from 1.0% in 1997 to 3.4% in 2017, suggesting that new therapies for cancer treatment have altered this risk^2^. VTE is considered the second most frequent cause of death in people with cancer, in addition to being responsible for greater risks of bleeding complications during anticoagulant treatment and recurrent venous thrombosis, than in people without malignant neoplasia^3^.

In this sense, cancer and the various treatments are recognized as independent risk factors for the development of VTE. The clinical association between cancer and hypercoagulability has been known for more than a century, and thromboembolic events are more frequent in people with cancer - one in five of them will present VTE during the natural course of the disease^2^.

There are several overlapping and interacting mechanisms that may explain the increased incidence of VTE in people with cancer. Cancer itself is associated with a four-fold increased risk of developing VTE, while antineoplastic chemotherapy increases this risk six-fold. People undergoing cytotoxic drug therapy are responsible for 13% of VTE episodes in the oncology population^2^.

Associated with these risks are the infections that affect the individual. In this context, the world recently faced a public health emergency with the COVID-19 pandemic (coronavirus disease 2019). COVID-19 is a disease caused by the virus called SARS-CoV-2, and with a high potential for contagion. The disease emerged in 2019 in China and spread quickly across the planet, causing respiratory symptoms that can be similar to a cold, flu or pneumonia^4^ ^)-(^ ^5^. COVID-19 is also considered a multisystemic disease, caused largely by the individual’s immune response and with predominantly endothelial involvement^6^.

Regarding the context of Venous Thromboembolism (VTE), COVID-19 is a disease that can cause hyperinflammation and has been associated with an increased risk of thromboembolic phenomena, especially pulmonary thromboembolism, more frequently observed in people with severe pneumonia, hospitalized in intensive care units^7^. A study conducted in France, in which 106 pulmonary angiograms were performed on people with COVID-19 over a period of one month, identified 32 people (30%) with acute pulmonary embolism. This rate of pulmonary embolism is much higher than what is typically found in people in critical care without COVID-19 infection (1.3%) or in people in emergencies (3 to 10%). As for arterial thrombosis, cases of acute ischemic stroke have been described in people with COVID-19, due to arterial obstruction of large vessels with a higher incidence than usual^6^.

During the COVID-19 pandemic, people with cancer were also affected by this new infection, adding another health risk to the chronic disease. Therefore, the importance of identifying the specificity of this population is observed, in order to guide assistance to people with cancer and care regarding the presence of risks for the development of thromboembolic events.

When considering the risk factors for thromboembolic events, recognized in people with cancer, and the thromboembolic events evidenced in people with COVID-19, this study aims to answer the following research question: people with cancer and a diagnosis of COVID-19 are at greater risk for develop thromboembolic events? Therefore, the objective of this study was to analyze the association between COVID-19 infection and thromboembolic events in people with cancer, during the first year of the pandemic.

## Method

### Study design

Case-control study with a ratio of 1 case/1 control in adults with cancer. This proportion was defined according to the time needed to carry out this research. The recommendations of the Strengthening the Reporting of Observational Studies in Epidemiology (STROBE)^8^ checklist were used to conduct and present the study.

### Setting

The study site was a reference oncology service in the public network in the state of Rio de Janeiro. It is a complex that brings together four units: 366 beds, including clinical hospitalization and intensive therapy, an outpatient network for consultations and imaging and laboratory tests, in addition to surgical center support. The choice of this scenario is justified because it is a reference health service in oncology, which also received people with cancer who were affected by COVID-19, during the pandemic.

### Period

Data collection was carried out between April 2021 and December 2022.

### Population

The cases were defined as: adults with cancer, regardless of oncological diagnosis, diagnosed with any type of thromboembolic event recorded in the medical record. Controls were defined as: adults with cancer, regardless of oncological diagnosis, without a diagnosis of a thromboembolic event. The following inclusion criteria were considered: being over 18 years old and being treated in any unit in the research scenario in the first year of the pandemic, between March 11, 2020, the initial date of the pandemic decreed by the World Health Organization^9^, and March 11, 2021, thus delimiting the first year of the pandemic. This time frame is justified by the changes that occurred in the second year of the pandemic, such as the beginning of vaccination and the change in the diagnostic method with the rapid test, which could interfere with the findings. Records that were not located or did not present information capable of providing the data necessary for the study were excluded, such as those that were incomplete, inconclusive or illegible.

### Sample definition

In order to identify the sample for this study, a prior search was carried out in the institution’s electronic medical records database for terms that suggested thromboembolic events in the period proposed for collection. In this way, 7,297 occurrences were found. By excluding duplicates - people with more than one occurrence, we reached a total of 2,986 records that had these terms. The occurrences were read and, of the total, 455 records contained conclusive reports of thromboembolic events. In this way, 455 records were also defined in the control group, totaling 910 records. After applying the exclusion criteria, the sample of interest with thromboembolic events consisted of 388 medical records (case group) and 440 for the control group, totaling 828 medical records included in the research.

**Figure 1:**
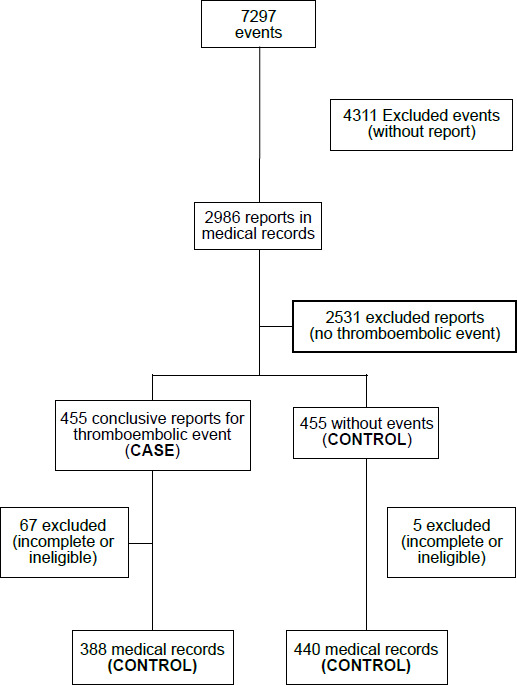
Selection of medical records for the study. Rio de Janeiro, RJ, Brazil, 2023

The pairing of samples between the case and control groups sought similarity between the individuals, so that the controls were similar to the cases in relation to certain characteristics other than those that deal with the factor under investigation. Thus, the definition of the control group in relation to the case group in this study occurred by comparing the variables sex and age, initially showing that the two groups were from a similar population, with regard to the distributions of these variables. For this, as the selected records were inserted into certain groups, partial reports were extracted, in order to verify the homogeneity of the groups in relation to these variables. When observing differences between groups, participants were excluded or included, in order to numerically balance them, according to the variables mentioned (Figure 1).

### Study variables

In this study, the diagnosis of any thromboembolic event - including arterial and venous - was considered as the outcome variable and the diagnosis of COVID-19 as the indicator variable to determine the subgroups. The other variables investigated were defined with the aim of characterizing people, according to their sociodemographic and clinical profile, in addition to identifying risk factors for thromboembolic events.

Variables aimed at characterizing the clientele were selected: age, marital status, gender, race/color, weight, height, Performance Status (PS); and clinical characteristics and risk factors for VTE: comorbidities, active cancer (yes or no), malignant neoplasm (yes or no), tumor site brain, pancreas, stomach, lung, bladder, gynecological, hematological and “others”), type treatment (surgery, antineoplastic chemotherapy, radiotherapy, etc.), tobacco use, immobility, laboratory test results, history of VTE, recent trauma, use of medications (oral contraceptives; erythropoiesis-stimulating agents), genetic factors - gene mutations Factor II - Prothrombin, Leiden Factor V^10^ or others - and score according to the Padua scale.

The Eastern Cooperative Oncology Group Performance Status Scale (PS-ECOG) assesses how the disease affects a person’s daily living skills, with scores ranging from zero to five points^11^. This variable was chosen because it is used in the institution where the study was carried out, recorded in the medical records, ideally carried out at each clinical evaluation. The Padua Score is a scale suggested by the American College of Chest Physicians (ACCP) as a way of assessing risk for VTE. This score evaluates the 14 risk factors, where each scored factor is added together to generate a cumulative risk. The final score defines the individual’s level of VTE risk, with a score ≥ 4 being high risk and a score < 4 being low risk^12^. These scales were not validated specifically for the population of this study and were used as variables because their data is available in medical records and their measurement does not suggest any risk to the research participant.

### Instruments used to collect information

The data were collected from secondary sources, from medical records of people treated at the service during the period determined for this research. An online computerized form was used, hosted on a website with exclusive access to the research team created specifically for this study, which addresses sociodemographic and clinical data, prepared based on the variables previously described according to the literature. 

### Data collection 

The collection involved four research assistants: undergraduate students who have earned a scholarship so that they could be part of research projects such as this. They were previously trained on site by the main researcher to enter the data, which were stored in the database managed by the main researcher. Research assistants were distributed between data collection and double-checking in order to guarantee the veracity of the findings.

They were blinded to the objectives of this study, as well as to the research question, as a way of minimizing confounding bias, in an attempt to make some association between the answers. The choice to collect data from medical records considered minimizing the memory bias that sometimes occurs when a person tries to remember events that occurred during hospitalization. The use of professional records as a source of data constitutes a possible bias, since they constitute a work tool and not a rigorous collection of information for studies. In this case, the absence or error in recording some information may influence the findings. 

### Data processing and analysis

The final version of the database was transported from Microsoft Excel^®^ to Stata software version 16.0. In the descriptive analysis, the distribution of sociodemographic, nutritional, clinical information, lifestyle habits, treatment and occurrence of events was presented. For qualitative variables (diagnosis and type of thromboembolic event, diagnosis of COVID-19, comorbidities, malignant neoplasia, active cancer, tumor site, treatment, chemotherapy, recent surgery or trauma, previous VTE, smoking, use of medications, prolonged immobilization, use of central venous catheter, genetic factors and others), absolute numbers and frequencies were calculated. For the quantitative variables (age, weight, height, PS, platelets, hemoglobin, d-dimer and Padua Score), position and dispersion measurements were calculated. Pearson’s chi-square test of independence was applied to verify the presence of an association between risk factors, considered independent variables, and the outcome of interest, adopted in this study, such as the occurrence of a thromboembolic event of any type. Logistic regression techniques were applied to identify the odds ratio of the occurrence of a thromboembolic event in sample strata of interest, with “No” being considered as the reference category or the category that represents, according to the literature, the lowest risk or better outcome.

The significance level adopted throughout the analysis was 5% and tables were used to present the results. When observing missing data in some participants, a test was chosen. The database was tested for variables with missing data, considering the original structure with all participants and an alternative composition, in which participants with missing data were removed. The chi-square test showed that the patterns of association between independent variables and the dependent variable did not change, which allowed us to infer the low impact of missing data in terms of associations.

### Ethical aspects

All declarations and terms of responsibility of the researcher were presented as required by the institution where the study was carried out. The study was approved by the Research Ethics Committees of the institutions involved, according to numbers 4,486,636 and 4,509,083.

## Results

A total of 828 medical records were included. Participants were mostly female (65%) and white (68%). The average age of the participants was 58.2 (±14.8) years. The most prevalent comorbidity in participants was hypertension, which was present in 43.5%, followed by diabetes (18%). Other comorbidities were present in 39.4% of participants. The diagnosis of COVID-19 was confirmed in 11.59% of participants overall and the overall death rate was 35.51% (Table 1).

**Table 1:** Sociodemographic and clinical characterization of study participants (n = 828). Rio de Janeiro, RJ, Brazil, 2021-2022

Variables			n	%
Gender (n=828)				
Female			539	65,10
Male			289	34,90
Race (n=828)				
White			564	68,12
Brown			204	24,64
Black			58	7,0
Age over 70 years old (n=826)				
Yes			144	17.43
No			682	82.57
Smoking (n=828)				
Yes			148	17.87
No			680	82.13
BMI classification (n=760)				
Severe underweight			12	1.58
Moderate low weight			14	1.84
Low light weight			36	4.74
Adequate			307	40.39
Overweight			244	32.11
Obesity grade 1			127	16.71
Grade 2 obesity			35	4.60
Grade 3 obesity			12	1.57
Obesity (n=760)				
Yes			174	22,86
No			587	77,14
COVID-19				
Yes			96	11,59
No			732	88,41
Death				
Yes			294	35,51
No			534	64,49
Hipertension				
Yes			360	43,48
No			468	56,52
Diabetes Mellitus				
Yes			149	18,0
No			679	82,0
Performance status				
0			100	12,08
1			350	42,27
2			132	15,94
3			117	14,13
4			129	15,58
**Variable**	Mean (±SD*)	**Median (IIQ** ^†^ **)**	**Minimum**	**Maximum**
Age	58,22 (±14,8)	60 (72)	18	91

*Standard deviation; ^†^Interquartile range

Out of the total of participants, 90.5% had malignant neoplasia, 73.91% had active cancer and the most common tumor site was the gynecological site (17.1%), followed by the breast (16.06%). Metastasis was present in 24.6% of the sample. Of the 828 people, 135 had laboratory test results with D-dimer values. High rates of this marker were noted in the studied population, with an average of 5,006.66 ng/ml, reaching values of up to 33,441 ng/ml. The average hemoglobin values were 11.71 g/dL (n=815) and platelets pre-antineoplastic chemotherapy were 281.09 thousand/mm^3^ (n=541). Antineoplastic chemotherapy was the most frequently adopted treatment (68.2%), followed by surgery (43.7%) and radiotherapy (40.82%). Recent antioplastic chemotherapy was performed in 26.9% of participants (Table 2).

**Table 2:** Clinical characterization of the oncological disease and treatments of people with cancer (N = 810). Rio de Janeiro, RJ, Brazil, 2021-2022

Variables			n	%
Malignant neoplasm				
Yes			749	90,46
No			79	9,54
Active cancer				
Yes			612	73,91
No			216	26,09
Tumor site: gynecological				
Yes			142	17,15
No			686	82,85
Tumor site: hematological				
Yes			70	8,45
No			758	91,55
Tumor site: breast				
Yes			133	16,06
No			695	83,94
Metastasis				
Yes			204	24,64
No			624	75,36
Antineoplastic chemotherapy				
Yes			565	68,24
No			263	31,76
Radiotherapy				
Yes			338	40,82
No			490	59,18
Surgery				
Yes			362	43,72
No			466	56,28
Use of Central Venous Catheter				
Yes			104	12,56
No			724	87,44
**Variables**	Mean (±SD*)	**Median (IIQ** ^†^ **)**	**Minimum**	**Maximum**
Antineoplastic pre-chemotherapy platelets (n=541)	281,09 (±313,88)	245 (824)	4	533,30
Hemoglobin (n=815)	11,71 (±6,91)	11,6 (81,0)	3,1	12,7
D-dimer (n=135)	5.006,66 (±6.240,76)	2.988 (22.490)	155	33.441
Length of stay (n=326)	12,68 (±15,86)	7,5 (74)	1	160
Padua score (n=810)	3,43 (2,18)	3 (10)	1	11

*Standard deviation; ^†^Interquartile range

In relation to other therapeutic procedures also considered risk factors for thromboembolic events, 13.41% of participants underwent blood transfusions.

### Comparison between case and control groups

When considering only the case group, the most common event in the studied population was deep vein thrombosis (65.98%), followed by pulmonary embolism (PE) (6.96%) and cerebrovascular accident (CVA) (3.61%). The events disseminated intravascular coagulation and acute myocardial infarction (AMI) were present at a lower frequency, 0.26% (n=1) and 1.8% (n=7) respectively. Other events totaled 21.39%.

Regarding the clinical characteristics of the neoplasms, it was identified that the presence of malignant neoplasm and active cancer were more frequent in the case group, which infers that they are associated with the thromboembolic event. The chance of this event occurring in individuals with malignant neoplasia is 79% greater compared to those who do not have it, while in people with active cancer the chance of experiencing a thromboembolic event is three times greater compared to those who do not have active cancer. Regarding the Padua risk score, there was no significant association with the event in the studied population (Table 3).

**Table 3:** Association between clinical characteristics and occurrence of thromboembolic events of any type (n = 828). Rio de Janeiro, RJ, Brazil, 2021-2022

Variables	Group				OR*	CI95%^†^	p^‡^
	Case		Control				
	n	%	n	%			
Malignant neoplasm							
Yes	361	48,20	388	51,8	1,791	1,101-2,914	0,018
No	27	34,18	52	65,82	1	-	
Active cancer							
Yes	334	54,58	278	45,42	3,604	2,548-5,097	<0,001
No	54	25,0	162	75,0	1	-	
No	368	46,0	432	54,0	1	-	
Tumor site: gynecological							
Yes	78	54,93	64	45,07	1,478	1,028-2,125	0,034
No	310	45,19	376	54,81	1	-	
Tumor site: hematological							
Yes	19	27,14	51	72,86	0,392	0,227-0,677	0,001
No	369	48,68	389	51,32	1	-	
Tumor site: breast							
Yes	42	31,58	91	68,42	0,465	0,313-0,690	<0,001
No	346	49,78	349	50,22	1	-	
Padua score (N=723)							
Up to 3	185	49,47	189	50,53	1	-	0,571
Same or higher than 4	180	51,58	169	48,42	0,919	0,686; 1,23	

*Odds ratio; ^†^95% confidence interval; ^‡^Chi-square test

Regarding treatments carried out on people with cancer, it was identified, in the case group, in relation to the control group, that antineoplastic chemotherapy, recent antineoplastic chemotherapy, blood transfusions and endocrine therapy are associated with the occurrence of thromboembolic events. The chance of these events occurring in people undergoing antineoplastic chemotherapy was 65% higher, compared to those who did not undergo it, while in people undergoing recent antineoplastic chemotherapy, the chance of presenting a thromboembolic event was 78% higher compared to those who did not undergo antineoplastic chemotherapy (Table 4).

**Table 4:** Association between treatment characteristics and occurrence of thromboembolic events of any type (n = 828). Rio de Janeiro, RJ, Brazil, 2021-2022

Variable	Group				OR*	CI95%^†^	p^‡^
	Case		Control				
	n	%	n	%			
Antineoplastic chemotherapy							
Yes	287	50,8	278	49,2	1,655	1,228-2,231	0,001
No	101	38,4	162	61,6	1	-	
Endocrine therapy							
Yes	35	36,46	61	63,54	0,616	0,396-0,956	0,030
No	353	48,22	379	51,78	1	-	
Recent antineoplastic chemotherapy							
Yes	128	57,40	95	42,6	1,787	1,310-2,438	<0,001
No	260	42,98	345	57,02	1	-	
Transfusions							
Yes	63	56,76	48	43,24	1,583	1,057-2,369	0,025
No	325	45,33	392	54,67	1	-	
Hemoglobin (N=816)							
< 10	132	57,39	98	42,61	1,772	1,302-2,412	<0,001
≥ 10	253	43,17	333	56,83	1	-	
COVID-19							
Yes	41	42,71	55	57,29	0,827	0,538-1,270	0,386
No	347	47,4	385	52,6	1	-	
Use of Central Venous Catheter							
Yes	49	47,12	55	52,88	1,011	0,670; 1,527	0,955
No	339	46,82	385	53,18	1	-	
Death outcome							
Yes	180	61,22	326	61,05	2,474	1,847-3,314	<0,001
No	280	38,95	114	38,78	1	-	
Performance status							
0	36	36,0	64	64,0	1	-	<0,001
1	143	40,86	207	59,14	1,228	0,774-1,946	
2	58	43,94	74	56,06	1,393	0,817-2,376	
3	73	62,39	44	37,61	2,949	1,695-5,131	
4	78	60,47	51	39,53	2,718	1,584-4,664	

*Odds ratio; ^†^95% confidence interval; ^‡^Chi-square test

Regarding COVID-19, 42.71% of people with a confirmed diagnosis were in the case group, however the association between COVID-19 and the thromboembolic event did not show statistical significance. Of the people who died, 61.22% were part of the case group and this showed relevance from a statistical point of view between these two conditions. Likewise, of the people undergoing outpatient follow-up, 38% were part of the case group and people with performance status values 3 and 4 were more present in the case group, showing a statistically significant relationship between this indicator and the occurrence of these events (Table 4).

The occurrence of any type of thromboembolic event (TE) had a significant association with death, and the event that had the greatest correlation with this outcome is not the subject of this study. Of the people who had ET, 46.39% died. Furthermore, hemoglobin had a statistically significant association with the event. People with hemoglobin lower than 10 were more likely to develop thromboembolic events. Hemoglobin status (less than or greater than 10) is associated with the occurrence of a thromboembolic event. The odds ratio for the occurrence of TE was 77% higher in people with hemoglobin lower than 10 (95%CI: 1.302-2.412; p<0.001), as shown in Table 4.

## Discussion

The study explores the occurrence of thromboembolic events and the clinical characteristics of people with cancer in the context of the COVID-19 pandemic in Brazil and, by characterizing the clinical aspects of people with malignant neoplasms according to the occurrence or not of these events, considering the variables sex and age, it was observed that the case and control groups were homogeneous among themselves. Furthermore, death rates were not different between the groups, reinforcing the complexity of the oncological disease. As found in the current study, several types of cancer treatment are factors associated with a greater chance of thromboembolic events.

In the context of people with cancer who also have other comorbidities, the potential for an increased risk of complications in the studied population is clear. In this aspect, a study can be seen in the literature that used a composite measure to assess the need for intensive care (intensive care center - ICU and mechanical ventilation) and death as a measure of risk of severity of COVID-19 infection, and identified risk 79% higher in individuals with any comorbidity, including cancer, DM, hypertension, cardiovascular and cerebrovascular diseases, when compared to individuals without comorbidities. For people with two or more associated comorbidities, the risk was 2.5 times higher^13^.

Regarding the incidence of cancer types with a higher risk of developing thromboembolic events, in this research, a higher frequency of gynecological (17.15%), breast (16.06%) and hematological (8.45%) tumors was observed, respectively. In this sense, a prospective and observational study of 10,684 patients with thrombosis was identified in the literature. In this study, 1,075 patients had active cancer, and among the most prevalent sites were breast (10.6%) and gynecological tumors (10.3%)^14^. Breast cancer was also among the most common in a study carried out in California, with a rate of 12% among those who had thromboembolic events^15^. However, it is noteworthy that the reported incidence of cancer-associated venous thrombosis varies widely between different studies due to differences in determination and the underlying populations represented.

The chance of a thromboembolic event occurring in individuals with malignant neoplasia in this study was 79% higher compared to those who did not have it, according to the results of this study. Furthermore, people with active cancer were three times more likely to have a thromboembolic event compared to those without active cancer. This data can be confirmed in the literature, as it is associated with biological factors that cause the risk of thrombosis in people with cancer. These factors include thrombin activation and fibrin formation. This activation is done directly by the release of procoagulant factors and cytokines that are produced by tumor cells. Cytokines stimulate intact endothelial cells and monocytes to express tissue factor in the outer membrane, causing activation of the coagulation cascade^16^.

Regarding thromboembolic events and hemoglobin values, the findings of this research corroborate the literature, in which it is observed that people with cancer have a substantially higher risk for new and recurrent episodes of deep vein thrombosis, when compared to people without cancer^3^. Furthermore, people with hemoglobin levels lower than 10 were more likely to develop thromboembolic events. This data confirms this indicator as a risk factor for thromboembolic events, according to a validation study of the Khorana Risk Prediction Scale^17^.

Deep vein thrombosis was the event that prevailed in the studied population (65.98%), followed by pulmonary embolism (6.96%). According to the literature, mortality rates from deep vein thrombosis attributed to idiopathic causes are lower than those observed among people with cancer. Up to 20% of people with cancer will develop thromboembolic events and the risk of thrombosis increases within a few months (zero to three months), after the diagnosis of malignancy and with the presence of metastasis^16^. It is noteworthy, in the present study, that thromboembolic events were associated with death, being more relevant than COVID-19 infection in the studied population.

The incidence of use of central venous catheters in the population of this study was low and consequently thromboembolic events related to catheters were not as present in this population. This data correlates with the context of the pandemic, in which the health service in the study setting prioritized urgent surgical care, impacting the insertion of catheters. The handling of central venous catheters is a fundamental element in the management of cancer patients as it is an important route for their treatment. Catheter-associated thrombosis is frequently observed in patients with malignancies; however, despite being a common complication among these patients, objective information about its epidemiology, clinical evolution, prophylaxis and treatment strategies is still very limited in current literature^18^.

The population in this research presented test results with high D-dimer values. In the context of COVID-19, a state of hypercoagulability and hematological changes occur, which have been described in up to a third of people, with increased D-dimer levels being an important marker of unfavorable outcomes^19^. Given this, some retrospective series investigated the frequency of Venous Thromboembolism in people with COVID-19, identifying the presence of changes in up to 40% of people^20^.

In this study, the elevation of the D-dimer marker was not relevant in the occurrence of thromboembolic events. This condition is common in COVID-19, which makes it difficult to use in the investigation of other events, such as thrombosis, for example^19^. The global death rate was high (35.51%), considering the context of COVID-19. A study with people undergoing clinical treatment showed a rate of 12%, the highest among elderly people in the pandemic scenario^21^. However, the death rate in this study is closer to the death rates found in people with cancer, regardless of COVID-19 infection^22^.

Antineoplastic chemotherapy, recent antineoplastic chemotherapy, blood transfusions and endocrine therapy showed a significant association with the occurrence of thromboembolic events. It was observed that in this study, the chance of these events occurring in people who underwent antineoplastic chemotherapy was 65% higher compared to those who did not undergo it, while in people undergoing recent antineoplastic chemotherapy, the chance of presenting a thromboembolic event was 78% higher, compared to those who did not do it.

In relation to cancer treatment, several factors contribute to the thromboembolic event. Abnormalities in the vessel walls are consequences of damage caused by oncological disease, whether caused by antineoplastic chemotherapy, surgery or the use of a venous catheter. Furthermore, chemotherapy agents are also associated with hypercoagulation, due to the reduction in plasma levels of physiological anticoagulants and the cytotoxic effect of antineoplastic chemotherapy that increases tissue factor expression and procoagulant activity^16^.

In this context, this result is corroborated by a recent cohort, the COVID-19 and Cancer Consortium (CCC19), which included 1,629 people with cancer, hospitalized with COVID-19 and concluded that recent anticancer therapy, active cancer, high-risk cancer subtypes for VTE and ICU admission were associated with an increased risk of VTE and PE. In contrast, in this study, it was shown that pre-admission anticoagulant or antiplatelet therapy can reduce this risk^23^.

It is therefore observed that cancer and its respective treatments showed greater relevance in the face of COVID-19 infection in relation to thromboembolic events. In this sense, the assessment of risk factors for these events through strategic tools continues to be an important recommendation. To this end, risk assessment scales were validated for people undergoing surgical procedures, people undergoing antineoplastic treatment, hospitalized people, among many other clinical conditions to be considered^24^ ^)-(^ ^25^. All of these forms of assessment include cancer as one of the risk factors and it is up to the professional to choose the one that best suits the person under their care. It is noteworthy that in this study, the Padua score did not show a statistically significant association with the occurrence of thromboembolic events, which infers that for this population, the ideal would be to use another already validated scale. Currently, only the Khorana risk scale is validated for people with cancer, but only on an outpatient basis, without considering aspects related to hospitalization and surgeries, for example^17^.

In addition to risk assessments, other preventive strategies can be designed for people with cancer and the consequent risk of thromboembolic events. The use of compression stockings, encouragement of early ambulation, mobilization in bed and analysis of laboratory tests should be used in the routine of health professionals, whether doctors or nurses, in order to identify risks and warning signs in this population^26^. Another action of the multidisciplinary team is to provide guidance to this individual and family, given that antineoplastic chemotherapy, whether recent or not, was a factor that increased the chance of a thromboembolic event occurring and, considering that a considerable proportion of people with cancer undergoing antineoplastic treatment do so on an outpatient basis. , that is, they have this risk in homes, it is up to the healthcare team to advise on the warning signs that should be considered when seeking immediate healthcare services^26^.

Therefore, when considering the discoveries about COVID-19 regarding transmissibility mechanisms, the pathophysiology of the disease, treatments and prevention, protection and control measures, the experience of the pandemic revealed new findings. Facing the COVID-19 pandemic required dynamism and restructuring of services to respond to the needs of the population, highlighting new techno-assistance arrangements both in the scope of management and health care, which is a major challenge in favor of promoting the health of people^27^. For this reason, identifying the characteristics of these people becomes so relevant. Some classic signs and symptoms of COVID-19 may also be symptoms of complications from the oncological treatment itself or from cancer, requiring the professional to prepare an evidence-based risk assessment, so that specific actions can be implemented, focusing on the need. of the individual at that moment^19^.

The limitations of the study are related to the methodology of an observational and retrospective study. The period and restricted population can directly influence the results and confidence intervals. Furthermore, the various adaptations to health services implemented in the first year of the pandemic, related to the handling of people with COVID-19, may also have influenced the findings. Therefore, these should not be generalized to hospital centers with different characteristics and to the world population. Another limitation refers to the use of professional records as a source of data as they constitute a work tool and not a rigorous collection of information for studies. 

## Conclusion

The present study confirmed the hypothesis that COVID-19 infection did not increase the chance of thromboembolic events in people with cancer. For the population studied, the factors that were associated with these events were those related to the neoplastic disease and the respective treatments. Furthermore, the study contributed to the literature, taking into account the gap in clinical research in the area of nursing aimed at this population. The study findings can guide nurses and the entire healthcare team in planning care for the high complexity identified in the studied population, trying to prevent thromboembolic events. Clinical studies are suggested that evaluate measures to prevent these events in a similar population, in order to identify the impact of these measures on the occurrence of these events.
