# Supplementary material for: Thromboembolic events in people with cancer during the COVID-19 pandemic: case-control study
Source: Rev Lat Am Enfermagem. 2024 Aug 19;32:e4266. doi: 10.1590/1518-8345.7075.4266 (PMC11335067; doi:10.1590/1518-8345.7075.4266)
Supplement: Translation

# Translation: Eventos tromboembólicos em pessoas com câncer na pandemia da COVID-19: estudo caso-controle*

**DOI:** 10.1590/1518-8345.7075.4267

**Authors:** Vivian Cristina Gama Souza Lima, Paulo Jorge Pereira Alves, Patrícia dos Santos Claro Fuly

**Affiliations:** 1 Universidade Federal Fluminense, Escola de Enfermagem Aurora de Afonso Costa, Niterói, RJ, Brasil.; 2 Ministério da Saúde, Instituto Nacional do Câncer, Rio de Janeiro, RJ, Brasil.; 3 Universidade Católica Portuguesa, Porto, Portugal.

**Keywords:** Enfermagem Oncológica, Oncologia, COVID-19, Trombose, Neoplasias, Estudos de Casos e Controles

## Abstract

**Objetivo:**

analisar a associação entre a infecção por coronavírus e os eventos tromboembólicos em pessoas com câncer, durante o primeiro ano da pandemia.

**Método:**

estudo caso-controle realizado mediante coleta em prontuários. Os casos estudados foram de adultos com câncer, com diagnóstico de evento tromboembólico, atendidos nas unidades do serviço selecionado durante o primeiro ano da pandemia. O grupo controle contou com adultos com câncer sem diagnóstico de evento tromboembólico. Teste qui-quadrado de Pearson foi aplicado para verificar associação entre fatores de risco e o desfecho de técnicas de regressão logística foram aplicadas para identificar a razão de chance de ocorrência de evento tromboembólico.

**Resultados:**

388 casos e 440 controles foram incluídos no estudo (proporção 1/1). Prevaleceu o sexo feminino, raça branca, média de idade de 58,2 (±14,8) anos. A quimioterapia antineoplásica foi o tratamento mais utilizado e a doença por coronavírus foi identificada em 11,59% dos participantes. No grupo caso, trombose venosa profunda foi mais prevalente.

**Conclusão:**

o estudo confirmou a hipótese de que a infecção por doença por coronavírus não aumentou a chance de eventos tromboembólicos em pessoas com câncer. Para a população estudada, os fatores que tiveram associação com os eventos foram os relacionados ao câncer e seu tratamento.

## Destaques

**(1)** Trombose venosa profunda foi a mais prevalente na população estudada.

**(2)** Quimioterapia aumentou a chance de eventos tromboembólicos em 65%.

**(3)** Eventos tromboembólicos mostraram associação significativa com a alta taxa de óbito.

**(4)** A COVID-19 não aumentou o risco de evento tromboembólico em pessoas com câncer.

## Introdução

O câncer é um dos principais problemas de saúde pública no Brasil e no mundo. Engloba mais de 100 tipos de doenças e, juntamente com as doenças cardiovasculares, respiratórias e o Diabetes Mellitus (DM), compõe o grupo das Doenças Crônicas não Transmissíveis (DCNT) responsáveis por cerca de 70% de todas as mortes no mundo^1^.

A crescente incidência das doenças oncológicas e a complexidade do cuidado aos indivíduos acometidos por elas envolvem vários aspectos, dentre eles o risco para o desenvolvimento de tromboembolismo venoso (TEV). Sabe-se que há estreita relação entre a doença oncológica e TEV, com risco aumentado de essas pessoas desenvolverem evento tromboembólico quando comparados à população em geral. Um estudo de coorte feito na Espanha comparou as taxas de incidência de tromboembolismo em populações com e sem câncer nos anos entre 1997 e 2017. A incidência cumulativa de TEV 12 meses após o diagnóstico do câncer foi de 2,3% na coorte de câncer e 0,35% na coorte sem câncer. Além disso, a incidência de 12 meses na coorte de câncer aumentou de 1,0% em 1997 para 3,4% em 2017, sugerindo que as novas terapias para tratamento do câncer tenham alterado esse risco^2^. O TEV é considerado a segunda causa mais frequente de óbito em pessoas com câncer, além de ser responsável por maiores riscos de complicações hemorrágicas durante o tratamento anticoagulante e de trombose venosa recorrente, isso quando comparado a pessoas sem neoplasia maligna^3^.

Dessa forma, o câncer e os diversos tratamentos são reconhecidos como fatores de risco independentes para o desenvolvimento de TEV. A associação clínica entre câncer e hipercoagulabilidade é conhecida há mais de um século, e os eventos tromboembólicos são mais frequentes em pessoas com câncer - uma em cada cinco delas apresentará TEV durante a evolução natural da doença^2^.

Há vários mecanismos que se sobrepõem e interagem e podem explicar o aumento da incidência de TEV nas pessoas com câncer. O câncer, por si só, está associado a um risco quatro vezes maior de desenvolvimento do TEV, enquanto a quimioterapia antineoplásica aumenta esse risco em seis vezes. Pessoas em terapia com citotóxicos são responsáveis por 13% dos episódios de TEV na população oncológica^2^. 

Associadas a tais riscos estão as infecções que acometem o indivíduo. Nesse contexto, recentemente, o mundo enfrentou uma emergência de saúde pública com a pandemia da COVID-19, uma doença causada pelo vírus denominado SARS-CoV-2, com alto potencial de contágio. A doença surgiu em 2019 na China e se espalhou rapidamente pelo planeta, causando sintomas respiratórios que podem ser semelhantes a um resfriado, gripe ou pneumonia^4^ ^)-(^ ^5^. A COVID-19 também é considerada uma doença multissistêmica, ocasionada, em grande parte, pela resposta imune do indivíduo e com envolvimento predominantemente endotelial^6^.

No que tange ao contexto do TEV, a COVID-19 é uma doença que pode provocar hiperinflamação e foi associada ao risco aumentado de ocorrência de fenômenos tromboembólicos, especialmente o tromboembolismo pulmonar, mais frequentemente observado em pessoas com pneumonia grave, internadas em unidades de terapia intensiva^7^. Em um estudo realizado na França, no qual 106 angiografias pulmonares foram realizadas em pessoas com COVID-19 durante o período de um mês, identificaram-se 32 pessoas (30%) com embolia pulmonar aguda. Essa taxa de embolia pulmonar é muito maior do que geralmente é encontrada em pessoas que estão em cuidados críticos sem infecção por COVID-19 (1,3%) ou em pessoas em situações de emergência (3 a 10%). Quanto à trombose arterial, descreve-se, em pessoas com COVID-19, casos de acidente vascular cerebral isquêmico agudo, devido à obstrução arterial de grandes vasos com incidência maior do que o habitual^6^.

Durante a pandemia da COVID-19, pessoas com câncer também foram acometidas por essa nova infecção, somando-se ao quadro da doença crônica mais um risco para a saúde. Sendo assim, observa-se a importância de identificar a especificidade dessa população, de forma a nortear a assistência às pessoas com câncer e o cuidado frente à presença dos riscos para desenvolvimento de eventos tromboembólicos.

Ao considerar os fatores de risco de eventos tromboembólicos, reconhecidos em pessoas com câncer e os eventos tromboembólicos evidenciados em pessoas com COVID-19, este estudo pretende responder à seguinte questão de pesquisa: pessoas com câncer e diagnóstico de COVID-19 apresenta maior risco para desenvolver eventos tromboembólicos? Dessa forma, o objetivo deste estudo foi analisar a associação entre a infecção por COVID-19 e os eventos tromboembólicos em pessoas com câncer, durante o primeiro ano da pandemia. 

## Método

### Tipo de estudo

Estudo caso-controle com proporção de 1 caso/1 controle em pessoas adultas com câncer. A proporção foi definida conforme o tempo hábil para realização desta pesquisa. Foram utilizadas as recomendações do *checklist Strengthening the Reporting of Observational Studies in Epidemiology* (STROBE)^8^ para condução e apresentação do estudo.

### Local do estudo

O local de estudo foi um serviço de referência em oncologia da rede pública do estado do Rio de Janeiro. Trata-se de um complexo abarcando quatro unidades com 366 leitos, incluindo internação clínica e terapia intensiva, rede ambulatorial para consultas, exames de imagem e laboratoriais, além de suporte de centro cirúrgico. A escolha do cenário se justifica por ser um serviço de saúde referência em oncologia, que recebeu também pessoas com câncer acometidas pela COVID-19 durante a pandemia.

### Período

A coleta de dados foi realizada entre abril de 2021 e dezembro de 2022.

### População

Os casos foram definidos como: adultos com câncer, independente do diagnóstico oncológico, diagnosticados com qualquer tipo de evento tromboembólico registrado em prontuário. Definiu-se como controles: adultos com câncer, independente do diagnóstico oncológico, sem diagnóstico de evento tromboembólico. Foram considerados os seguintes critérios de inclusão: ter idade a partir de 18 anos e ser atendido em uma das unidades do cenário de pesquisa no primeiro ano da pandemia, compreendido entre 11 de março de 2020, data inicial da pandemia decretada pela Organização Mundial de Saúde^9^, e 11 de março de 2021, delimitando, assim, o primeiro ano da pandemia. Esse recorte temporal se justifica pelas mudanças que ocorreram no segundo ano da pandemia como o início da vacinação e a mudança no método diagnóstico com o teste rápido, os quais poderiam interferir nos achados. Excluíram-se os prontuários que não foram localizados ou não apresentaram informações para fornecer os dados necessários ao estudo, como os incompletos, inconclusivos ou ilegíveis.

### Definição da amostra

Para identificar a amostra realizou-se busca prévia na base eletrônica de prontuários da instituição por termos que sugerissem os eventos tromboembólicos no período proposto para coleta. Nela, encontrou-se 7.297 ocorrências. Ao excluir as duplicatas - pessoas com mais de uma ocorrência, chegou-se ao total de 2.986 prontuários que possuíam esses termos. Procedeu-se à leitura das ocorrências e, do total, 455 prontuários continham laudos conclusivos de eventos tromboembólicos. Assim, definiu-se 455 prontuários também no grupo controle, totalizando 910 prontuários. Após aplicação dos critérios de exclusão, a amostra de interesse com evento tromboembólico foi composta por 388 prontuários (grupo caso) e de 440 para o grupo controle, totalizando 828 prontuários incluídos na pesquisa. 

Para o pareamento das amostras entre os grupos caso e controle buscou-se a semelhança entre os indivíduos, de forma que os controles fossem semelhantes aos casos em relação a certas características que não aquelas que tratam do fator sob investigação. Desse modo, a definição do grupo controle em relação ao grupo caso, neste estudo, ocorreu pela comparação entre as variáveis sexo e idade, mostrando, inicialmente, que os dois grupos eram de população semelhante, no que se refere a distribuição dessas variáveis. Conforme os prontuários selecionados eram inseridos nos determinados grupos, os relatórios parciais eram extraídos, a fim de verificar a homogeneidade dos grupos em relação às variáveis. Ao observar a diferença entre os grupos, os participantes eram excluídos ou incluídos, a fim de equilibrar numericamente os mesmos, conforme Figura 1.

**Figura 1:** Seleção dos prontuários para o estudo. Rio de Janeiro, RJ, Brasil, 2023

### Variáveis do estudo

Neste estudo, considerou-se o diagnóstico de qualquer evento tromboembólico - incluindo arteriais e venosos - como variável de desfecho e o diagnóstico de COVID-19 como variável indicadora para determinação dos subgrupos. As demais variáveis investigadas foram definidas com o objetivo de caracterizar as pessoas, de acordo com o perfil sociodemográfico e clínico, além de identificar os fatores de risco para eventos tromboembólicos.

Foram selecionadas as variáveis demograficas: idade, estado civil, sexo, raça/cor, peso, altura, Performance Status (PS); clínicas e fatores de risco para TEV: comorbidades, câncer ativo (sim ou não), neoplasia maligna (sim ou não), sítio tumoral cérebro, pâncreas, estômago, pulmão, bexiga, ginecológicos, hematológicos, dentre outros), tipo de tratamento (cirurgia, quimioterapia antineoplásica, radioterapia, entre outros), uso de tabaco, imobilidade, resultados de exames laboratorias, histórico de TEV, traumas recentes, uso de medicamentos (anticoncepcional oral; agentes estimulantes da eritropoiese), fatores genéticos - mutações dos genes Fator II - Protrombina, Fator V de Leiden^10^ ou outros - e escore conforme escala de Pádua.

A Escala de *Performance Status* do *Eastern Cooperative Oncology Group* (PS-ECOG) permite avaliar como a doença afeta as habilidades de vida diária da pessoa, com escore que varia de zero a cinco pontos^11^. Optou-se por esta escala, pois ela é utilizada na instituição cenário do estudo, com registro em prontuário, a cada avaliação clínica. 

O Escore de Pádua é uma escala sugerida pela *American College of Chest Physicians* (ACCP), que permite a avaliação de risco para TEV. O escore avalia os 14 fatores de risco, em que cada fator pontuado é somado para gerar um risco cumulativo. A pontuação final define o nível de risco de TEV do indivíduo, sendo escore ≥ 4 alto risco e escore < 4 baixo risco^12^. As escalas não foram validadas especificamente para a população deste estudo e foram utilizadas para obtenção de variáveis disponíveis em prontuário e sua mensuração não sugere risco ao participante da pesquisa.

### Instrumento utilizados para a coleta de dados

Os dados foram coletados a partir de fontes secundárias, isto é, os prontuários das pessoas atendidas no serviço no recorte temporal definido para esta pesquisa. Utilizou-se de formulário informatizado *online*, hospedado em site de acesso exclusivo da equipe de pesquisa criado especificamente para este estudo, contendo as variáveis clínicas, elaborado a partir das variáveis eleitas para o estudo.

### Coleta de dados

A coleta contou com quatro assistentes de pesquisa, que participaram como bolsistas de iniciação científica no projeto de pesquisa, previamente treinados *in loco* pela pesquisadora principal para inserção dos dados, os quais ficaram armazenados no banco de dados gerenciado pela pesquisadora principal. Os assistentes de pesquisa se distribuíram entre coleta e dupla checagem dos dados a fim de garantir a veracidade dos achados. Eles foram cegados para os objetivos deste estudo tanto quanto para a questão de pesquisa, como forma de minimizar viés de confusão, na tentativa de realizar alguma associação entre as respostas. A escolha pela coleta em prontuários considerou minimizar o viés de memória que, por vezes, ocorre quando uma pessoa tenta lembrar dos fatos ocorridos durante a internação hospitalar. O uso de registros profissionais como fonte de dados se configura como possível viés, uma vez que constituem ferramenta de trabalho e não de coleta rigorosa de informação para estudos. Nesse caso, a ausência ou o erro de registro de algumas informações podem influenciar os achados. 

### Tratamento e análise do dados

A versão final do banco de dados foi transportada do Microsoft Excel^®^ para o *software* Stata versão 16.0. Na análise descritiva, procedeu-se à apresentação da distribuição das informações sociodemográficas, nutricionais, clínicas, de hábitos de vida, de tratamento e ocorrência de eventos. Para as variáveis qualitativas (diagnóstico e tipo de evento tromboembólico, diagnóstico de COVID-19, comorbidades, neoplasia maligna, câncer ativo, sítio tumoral, tratamento, quimioterapia, cirurgia ou trauma recente, TEV prévio, tabagismo, uso de medicamentos, imobilização prolongada, uso de cateter venoso central, fatores genéticos e outros), calcularam-se os números absolutos e as frequências. Para as variáveis quantitativas (idade, peso, altura, PS, plaquetas, hemoglobina, d-dímero e Escore de Pádua), calculou-se medidas de posição e dispersão. O teste de independência, do tipo qui-quadrado de Pearson, foi aplicado para verificar presença de associação entre fatores de risco, considerados como variáveis independentes, e o desfecho de interesse, adotado, neste estudo, como a ocorrência de evento tromboembólico de qualquer tipo. Técnicas de regressão logística foram aplicadas para identificar a razão de chance de ocorrência de evento tromboembólico em estratos amostrais de interesse, sendo considerada como categoria de referência o “Não” ou a categoria que representa, de acordo com a literatura, o menor risco ou o melhor desfecho. 

O nível de significância adotado em toda a análise foi de 5% e utilizaram-se de tabelas para apresentar os resultados. Ao observar dados ausentes em alguns participantes, optou-se por um teste. O banco de dados foi testado para as variáveis com dados ausentes, considerando a estrutura original com todos os participantes e uma composição alternativa, na qual foram retirados os participantes com dados ausentes. O teste qui-quadrado mostrou que os padrões de associação entre variáveis independentes e a variável dependente não mudaram, o que permitiu inferir a baixa repercussão dos dados ausentes no aspecto das associações.

### Aspectos éticos

Foram apresentadas todas as declarações e termos de responsabilidade do pesquisador conforme exigência da instituição cenário do estudo. O estudo foi aprovado pelos Comitês de Ética em Pesquisa das instituições envolvidas, conforme os pareceres números 4.486.636 e 4.509.083. 

## Resultados

Foram incluídos 828 prontuários no total. Os participantes foram majoritariamente do sexo feminino (65%) e de raça branca (68%). A média de idade dos participantes foi de 58,2 (±14,8) anos. A comorbidade mais prevalente foi a hipertensão arterial a qual esteve presente em 43,5%, seguida por DM (18%). Outras comorbidades estavam presentes em 39,4% dos participantes. O diagnóstico de COVID-19 foi confirmado em 11,59% dos participantes no geral e a taxa de óbito global foi de 35,51% (Tabela 1).

**Tabela 1:** Caracterização sociodemográfica e clínica dos participantes do estudo (n = 828). Rio de Janeiro, RJ, Brasil, 2021-2022

Variáveis			n	%
Sexo (n=828)				
Feminino			539	65,10
Masculino			289	34,90
Raça (n=828)				
Branca			564	68,12
Parda			204	24,64
Preta			58	7,0
Idade superior a 70 anos (n=826)				
Sim			144	17.43
Não			682	82.57
Tabagismo (n=828)				
Sim			148	17.87
Não			680	82.13
Classificação de IMC (n=760)				
Baixo peso severo			12	1.58
Baixo peso moderado			14	1.84
Baixo peso leve			36	4.74
Adequado			307	40.39
Sobrepeso			244	32.11
Obesidade grau 1			127	16.71
Obesidade grau 2			35	4.60
Obesidade grau 3			12	1.57
Obesidade (n=760)				
Sim			174	22,86
Não			587	77,14
COVID-19				
Sim			96	11,59
Não			732	88,41
Óbito				
Sim			294	35,51
Não			534	64,49
Hipertensão Arterial				
Sim			360	43,48
Não			468	56,52
Diabetes Mellitus				
Sim			149	18,0
Não			679	82,0
Performance status				
0			100	12,08
1			350	42,27
2			132	15,94
3			117	14,13
4			129	15,58
**Variável**	Média (±DP*)	**Mediana (IIQ** ^†^ **)**	**Mínimo**	**Máximo**
Idade	58,22 (±14,8)	60 (72)	18	91

*Desvio-padrão; ^†^Intervalo interquartílico

Do total de participantes, 90,5% apresentaram neoplasia maligna, 73,91% tinham câncer ativo e o sítio tumoral mais frequente foi o ginecológico (17,1%), seguido da mama (16,06%). Metástase estava presente em 24,6% da amostra. Das 828 pessoas, 135 possuíam resultados de exames laboratoriais com valores de D-dímero. Notou-se elevadas taxas deste marcador na população estudada, sendo a média de 5.006,66 ng/ml, chegando a valores de até 33.441 ng/ml. Os valores médios de hemoglobina ficaram em 11,71 g/dL (n=815) e de plaquetas pré-quimioterapia antineoplásica 281,09 mil/mm^3^ (n=541). Quimioterapia antineoplásica foi o tratamento mais frequentemente adotado (68,2%), seguida por cirurgia (43,7%) e radioterapia (40,82%). Quimioterapias antioplásicas recentes foram realizadas em 26,9% dos participantes (Tabela 2).

**Tabela 2:** Caracterização clínica da doença oncológica e tratamentos das pessoas com câncer (n = 810). Rio de Janeiro, RJ, Brasil, 2021-2022

Variáveis		n	%	
Neoplasia maligna				
Sim		749	90,46	
Não		79	9,54	
Câncer ativo				
Sim		612	73,91	
Não		216	26,09	
Sítio tumoral: ginecológico				
Sim		142	17,15	
Não		686	82,85	
Sítio tumoral: hematológico				
Sim		70	8,45	
Não		758	91,55	
Sítio tumoral: mama				
Sim		133	16,06	
Não		695	83,94	
Metástase				
Sim		204	24,64	
Não		624	75,36	
Quimioterapia antineoplásica				
Sim		565	68,24	
Não		263	31,76	
Radioterapia				
Sim		338	40,82	
Não		490	59,18	
Cirurgia				
Sim		362	43,72	
Não		466	56,28	
Uso de Cateter Venoso Central				
Sim		104	12,56	
Não		724	87,44	
**Variáveis**	Média (±DP*)	**Mediana (IIQ** ^†^)	**Mínimo**	**Máximo**
Plaquetas pré-quimioterapia antineoplásica (N=541)	281,09 (±313,88)	245 (824)	4	533,30
Hemoglobina (N=815)	11,71 (±6,91)	11,6 (81,0)	3,1	12,7
D-dímero (N=135)	5.006,66 (±6.240,76)	2.988 (22.490)	155	33.441
Tempo de internação (N=326)	12,68 (±15,86)	7,5 (74)	1	160
Escore Pádua (N=810)	3,43 (2,18)	3 (10)	1	11

*Desvio-padrão; ^†^Intervalo interquartílico

Em relação a outros procedimentos terapêuticos também considerados fatores de risco para eventos tromboembólicos, 13,41% dos participantes realizaram hemotransfusões. 

### Comparação entre os grupos caso e controle

Ao considerar apenas o grupo caso, o evento mais frequente na população estudada foi a trombose venosa profunda (65,98%), seguido da embolia pulmonar (EP) (6,96%) e do acidente vascular cerebral (AVC) (3,61%). Os eventos coagulação intravascular disseminada e infarto agudo do miocárdio (IAM) estiveram presentes em menor frequência, 0,26% (n=1) e 1,8% (n=7) respectivamente. Outros eventos somaram 21,39%.

Em relação às características clínicas das neoplasias, identificou-se que a presença de neoplasia maligna e de câncer ativo estavam em maior ocorrência no grupo caso, o que infere que estejam associados ao evento tromboembólico. A chance de ocorrência desse evento em indivíduos com neoplasia maligna é 79% maior em comparação àqueles que não a possuem, enquanto em pessoas com câncer ativo a chance de apresentação do evento tromboembólico é três vezes maior em comparação a quem não possui câncer ativo. Quanto ao escore de risco de Pádua, não houve associação significativa com o evento na população estudada (Tabela 3).

**Tabela 3:** Associação entre características clínicas e ocorrência de evento tromboembólico de qualquer tipo (n = 828). Rio de Janeiro, RJ, Brasil, 2021-2022

Variáveis	Grupo				OR*	IC95%^†^	p^‡^
	Caso		Controle				
	n	%	n	%			
Neoplasia maligna							
Sim	361	48,20	388	51,8	1,791	1,101-2,914	0,018
Não	27	34,18	52	65,82	1	-	
Câncer ativo							
Sim	334	54,58	278	45,42	3,604	2,548-5,097	<0,001
Não	54	25,0	162	75,0	1	-	
Não	368	46,0	432	54,0	1	-	
Sítio tumoral: ginecológico							
Sim	78	54,93	64	45,07	1,478	1,028-2,125	0,034
Não	310	45,19	376	54,81	1	-	
Sítio tumoral: hematológico							
Sim	19	27,14	51	72,86	0,392	0,227-0,677	0,001
Não	369	48,68	389	51,32	1	-	
Sítio tumoral: mama							
Sim	42	31,58	91	68,42	0,465	0,313-0,690	<0,001
Não	346	49,78	349	50,22	1	-	
Escore de Pádua (N=723)							
Até 3	185	49,47	189	50,53	1	-	0,571
Igual ou maior a 4	180	51,58	169	48,42	0,919	0,686; 1,23	

* *Odds ratio* (Razão de chances); ^†^Intervalo de confiança de 95%; ^‡^Teste qui-quadrado

No que se refere aos tratamentos realizados em pessoas com câncer, identificou-se, no grupo caso em relação ao controle, que a realização de quimioterapia antineoplásica, quimioterapia antineoplásica recente, transfusões sanguíneas e terapia endócrina estão associados à ocorrência de evento tromboembólico. A chance de ocorrência desses eventos em pessoas que realizam quimioterapia antineoplásica foi 65% maior em comparação àqueles que não a realizam, enquanto que, em pessoas em quimioterapia antineoplásica recente, a chance de apresentação do evento tromboembólico foi 78% superior em comparação a quem não a realizava (Tabela 4).

**Tabela 4:** Associação entre características de tratamento e ocorrência de evento tromboembólico de qualquer tipo (n = 828). Rio de Janeiro, RJ, Brasil, 2021-2022

Variáveis	Grupo				OR*	IC95%^†^	p^‡^
	Caso		Controle				
	n	%	n	%			
Quimioterapia antineoplásica							
Sim	287	50,8	278	49,2	1,655	1,228-2,231	0,001
Não	101	38,4	162	61,6	1	-	
Terapia endócrina							
Sim	35	36,46	61	63,54	0,616	0,396-0,956	0,030
Não	353	48,22	379	51,78	1	-	
Quimioterapia antineoplásica recente							
Sim	128	57,40	95	42,6	1,787	1,310-2,438	<0,001
Não	260	42,98	345	57,02	1	-	
Transfusões							
Sim	63	56,76	48	43,24	1,583	1,057-2,369	0,025
Não	325	45,33	392	54,67	1	-	
Hemoglobina (n=816)							
< 10	132	57,39	98	42,61	1,772	1,302-2,412	<0,001
≥ 10	253	43,17	333	56,83	1	-	
COVID-19							
Sim	41	42,71	55	57,29	0,827	0,538-1,270	0,386
Não	347	47,4	385	52,6	1	-	
Uso de Cateter Venoso Central							
Sim	49	47,12	55	52,88	1,011	0,670; 1,527	0,955
Não	339	46,82	385	53,18	1	-	
Desfecho óbito							
Sim	180	61,22	326	61,05	2,474	1,847-3,314	<0,001
Não	280	38,95	114	38,78	1	-	
*Performance status*							
0	36	36,0	64	64,0	1	-	<0,001
1	143	40,86	207	59,14	1,228	0,774-1,946	
2	58	43,94	74	56,06	1,393	0,817-2,376	
3	73	62,39	44	37,61	2,949	1,695-5,131	
4	78	60,47	51	39,53	2,718	1,584-4,664	

*Odds ratio (Razão de chances); ^†^Intervalo de confiança de 95%; ^‡^Teste qui-quadrado

Quanto à COVID-19, 42,71% das pessoas com diagnóstico confirmado estavam no grupo caso, porém a associação entre a COVID-19 e o evento tromboembólico não mostrou significância estatística. Das pessoas que foram a óbito, 61,22% faziam parte do grupo caso e isso mostrou relevância, do ponto de vista estatístico, entre estas duas condições. Da mesma forma, das pessoas em acompanhamento ambulatorial, 38% faziam parte do grupo caso, pessoas com valores de *performance status* 3 e 4 estavam mais presentes no grupo caso, mostrando relação estatisticamente significativa deste indicador com a ocorrência dos referidos eventos (Tabela 4).

A ocorrência de qualquer tipo de evento tromboembólico (ET) teve associação significativa com o óbito, não sendo objeto deste estudo qual evento que teve maior correlação com este desfecho. Das pessoas que tiveram ET, 46,39% foram a óbito. Além disso, a hemoglobina teve associação estatística significativa com evento. Pessoas com hemoglobina menor que 10 tiveram maior chance para desenvolver eventos tromboembólicos**.** A condição da hemoglobina (menor ou maior que 10) está associada à ocorrência de evento tromboembólico. A razão de chance para ocorrência de ET foi 77% maior em pessoas com hemoglobina e menor do que 10 (IC95%: 1,302-2,412; p<0,001), conforme Tabela 4.

## Discussão

O estudo explora a ocorrência de eventos tromboembólicos e as características clínicas de pessoas com câncer no contexto da pandemia da COVID-19 no Brasil. Ao caracterizar os aspectos clínicos de pessoas com neoplasias malignas de acordo com a ocorrência ou não desses eventos, considerando as variáveis sexo e idade, observou-se que os grupos caso e controle foram homogêneos entre si. Além disso, as taxas de óbitos não foram diferentes entre os grupos, reforçando a complexidade da doença oncológica. Como achado no estudo em tela, têm-se diversos tipos de tratamento para o câncer como fatores associados a maior chance de ocorrência de eventos tromboembólicos. 

No contexto de pessoas com câncer e comorbidades, constata-se o potencial para aumento do risco de complicações na população estudada. Nesse aspecto, um estudo que utilizou a medida composta para avaliar a necessidade de cuidados intensivos (centro de terapia intensiva - CTI e ventilação mecânica) e óbito como medida de risco de gravidade da infecção por COVID-19, identificou risco 79% maior em indivíduos com comorbidade, incluindo câncer, DM, hipertensão arterial, doenças cardiovasculares e cerebrovasculares, quando comparados a indivíduos sem comorbidades. Para pessoas com duas ou mais comorbidades associadas, o risco foi 2,5 vezes mais elevado^13^.

Quanto à incidência dos tipos de câncer com maior risco para desenvolvimento de eventos tromboembólicos, observou-se, nesta pesquisa, maior frequência de tumores ginecológicos (17,15%), mama (16,06%) e hematológico (8,45%), respectivamente. Nesse sentido, identificou-se na literatura um estudo prospectivo e observacional de 10.684 pacientes com trombose. Neste estudo, 1.075 pacientes possuíam câncer ativo e, dentre os sítios mais prevalentes, observou-se mama (10,6%) e tumores ginecológicos (10,3%)^14^. O câncer de mama também esteve entre os mais frequentes em estudo realizado, na Califórnia, com taxa de 12% dentre os que tiveram eventos tromboembólicos^15^. No entanto, salienta-se que a incidência relatada de trombose venosa associada ao câncer varia amplamente entre os diversos estudos devido a diferenças na determinação e nas populações subjacentes representadas.

A chance de ocorrência de evento tromboembólico em indivíduos com neoplasia maligna foi 79% maior em comparação àqueles que não a possuem, segundo os resultados deste estudo. Ademais, pessoas com câncer ativo apresentaram três vezes mais chance de evento tromboembólico em comparação a quem não possui câncer ativo. Tal dado pode ser confirmado na literatura, pois está associado aos fatores biológicos que ocasionam o risco de trombose em pessoas com câncer. Esses fatores incluem a ativação da trombina e a formação de fibrina. A ativação se faz diretamente pela liberação de fatores pró-coagulantes e citocinas que são produzidas pelas células tumorais. As citocinas estimulam as células endoteliais íntegras e os monócitos a expressarem o fator tecidual na membrana externa, acarretando ativação da cascata de coagulação^16^.

Quanto aos eventos tromboembólicos e valores de hemoglobina, os achados desta pesquisa corroboram com a literatura, na qual se observa que as pessoas com câncer têm risco substancialmente mais elevado para episódios novos e recorrentes de trombose venosa profunda, quando comparados a pessoas sem câncer^3^. Além disso, pessoas com hemoglobina menor do que 10 tiveram maior chance para desenvolver eventos tromboembólicos. O dado confirma esse indicador como fator de risco para eventos tromboembólicos, conforme estudo de validação da Escala de Predição de Risco de Khorana^17^. 

A trombose venosa profunda foi o evento que prevaleceu na população estudada (65,98%), seguida da embolia pulmonar (6,96%). Consoante com a literatura, as taxas de mortalidade por trombose venosa profunda atribuídas às causas idiopáticas são mais baixas do que as observadas entre as pessoas com câncer. Até 20% das pessoas com câncer desenvolverão eventos tromboembólicos e o risco de trombose aumenta em poucos meses (zero a três meses), após o diagnóstico de malignidade e com a presença de metástase^16^. Destaca-se, no estudo em tela, que os eventos tromboembólicos estiveram associados com o óbito, sendo mais relevantes do que a infecção por COVID-19 na população estudada. 

A incidência de uso de cateteres venosos centrais na população deste estudo apresentou-se baixa e consequentemente os eventos tromboembólicos relacionados a cateres não estiveram tão presentes nesta população. O dado correlaciona-se com o contexto da pandemia, no qual o serviço de saúde cenário do estudo priorizou atendimentos cirúrgicos de urgência, impactando a inserção de cateteres. O manuseio de cateteres venosos centrais é um elemento fundamental no manejo do paciente com câncer por ser uma via importante para seu tratamento. A trombose associada a cateter é frequentemente observada em pacientes com doenças malignas; entretanto, apesar de ser uma complicação comum entre esses pacientes, as informações objetivas sobre sua epidemiologia, evolução clínica, profilaxia e estratégias de tratamento ainda são muito limitadas na literatura atual^18^.

A população desta pesquisa apresentou resultados de exames com valores altos de D-dímero. No contexto da COVID-19, ocorre estado de hipercoagulabilidade e alterações hematológicas, os quais têm sido descritos em até um terço das pessoas, sendo a elevação dos níveis de D-dímero um importante marcador de desfechos desfavoráveis^19^. Diante disso, algumas séries retrospectivas investigaram a frequência de Tromboembolismo Venoso em pessoas com COVID-19, identificando a presença de alterações em até 40% das pessoas^20^.

Neste estudo, a elevação do marcador D-dímero não se mostrou relevante na ocorrência de eventos tromboembólicos. Esta condição é comum na COVID-19, o que dificulta a utilização na investigação de outros eventos, como a trombose por exemplo^19^. Já a taxa de óbito global mostrou-se alta (35,51%), considerando o contexto da COVID-19. Um outro estudo desenvolvido com pessoas em tratamento clínico evidenciou a taxa de 12%, a mais elevada em idosos no cenário da pandemia^21^. No entanto, a taxa de óbito deste estudo se aproxima mais das taxas de óbito evidenciadas em pessoas com câncer, independente da infecção por COVID-19^22^.

A realização de quimioterapia antineoplásica, quimioterapia antineoplásica recente, transfusões sanguíneas e terapia endócrina mostraram associação significativa com a ocorrência de evento tromboembólico. Observou-se que, neste estudo, a chance de ocorrência desses eventos em pessoas que realizaram quimioterapia antineoplásica foi 65% maior em comparação àqueles que não a realizaram, enquanto em pessoas em quimioterapia antineoplásica recente, a chance de apresentação do evento tromboembólico foi 78% superior em comparação a quem não a realizou. 

Em relação ao tratamento do câncer, diversos fatores contribuem para o evento tromboembólico. Anormalidades das paredes dos vasos são consequências dos danos da doença oncológica, seja pela quimioterapia antineoplásica, seja pelas cirurgias ou pelo uso de cateter venoso. Além disso, os agentes quimioterápicos também estão associados à hipercoagulação, pela redução de níveis plasmáticos de anticoagulantes fisiológicos e pelo efeito citotóxico da quimioterapia antineoplásica que aumenta a expressão do fator tecidual e a atividade pró-coagulante^16^. 

Nesse contexto, o resultado é corroborado por coorte recente, a *COVID-19 and Cancer Consortium* (CCC19), que incluiu 1.629 pessoas com câncer, hospitalizadas com COVID-19 e concluiu que terapia anticancerígena recente, câncer ativo, subtipos de câncer com alto risco para TEV e internação em CTI foram associados ao risco aumentado de TEV e EP. Em contraste, neste estudo, evidenciou-se que a terapia anticoagulante ou antiplaquetária pré-admissão pode reduzir esse risco^23^. 

Observa-se, dessa forma, que o câncer e os respectivos tratamentos mostraram maior relevância diante da infecção por COVID-19 em relação aos eventos tromboembólicos. Dessa forma, a avaliação dos fatores de risco para esses eventos através de ferramentas estratégicas continua sendo uma recomendação importante. Para isso, escalas de avaliação de risco foram validadas para pessoas submetidas a procedimentos cirúrgicos, pessoas em vigência de tratamento antineoplásico, pessoas hospitalizadas, dentre tantas outras condições clínicas a serem consideradas^24^ ^)-(^ ^25^. Todas essas formas de avaliação incluem o câncer como um dos fatores de risco e cabe ao profissional eleger aquela que mais se adequa à pessoa sob seus cuidados. Ressalta-se que, neste estudo, o escore de Pádua não mostrou associação estatística significativa na ocorrência de eventos tromboembólicos, o que infere que para essa população, o ideal seja usar outra escala já validada. Atualmente, apenas a escala de risco de Khorana está validada para pessoas com câncer, porém apenas a nivel ambulatorial, sem considerar aspectos relacionados a hospitalização e cirurgias por exemplo^17^.

Além das avalições de risco, pode-se traçar outras estratégias preventivas as pessoas com câncer e consequente risco para evento tromboembólico. O uso de meias de compressão, estímulo à deambulação precoce, mobilização no leito e análise de exames laboratoriais devem ser empregados na rotina do profissional de saúde, seja médico ou enfermeiro, a fim de identificar riscos e sinais de alerta nessa população^26^. Outra ação da equipe multidisciplinar é a orientação ao indivíduo e à família, sobretudo tendo em vista que a quimioterapia antineoplásica, recente ou não, foi um fator que aumentou a chance de ocorrência do evento tromboembólico e, levando em conta que uma parte considerável das pessoas com câncer em tratamento antineoplásico o fazem ambulatorialmente, ou seja, possuem esse risco em residências, cabe à equipe de saúde a orientação quanto aos sinais de alerta que devem ser considerados para procura imediata do serviço de saúde^26^.

Dessa forma, ao ponderar as descobertas sobre a COVID-19 quanto aos mecanismos de transmissibilidade, à fisiopatologia da doença, aos tratamentos e às medidas de prevenção, proteção e controle, a experiência da pandemia revelou novos achados. O enfrentamento da pandemia de COVID-19 exigiu dinamicidade e reestruturação dos serviços para responder às necessidades da população, evidenciando-se novos arranjos tecnoassistenciais tanto no âmbito da gestão quanto da atenção à saúde, sendo um grande desafio em prol da promoção da saúde das pessoas^27^. Por isso, identificar a característica dessas pessoas torna-se tão relevante. Alguns sinais e sintomas clássicos da COVID-19 podem ser também sintomas de complicações do próprio tratamento oncológico ou do adoecimento por câncer, exigindo do profissional a elaboração de avaliação de risco baseada em evidências, para que sejam implementadas as ações específicas, com foco na necessidade do indivíduo no momento^19^.

As limitações do estudo estão relacionadas à metodologia de um estudo observacional e retrospectivo. O período e a população restrita podem influenciar diretamente os resultados e intervalos de confiança. Além disso, as diversas adaptações dos serviços de saúde implementadas no primeiro ano da pandemia, relacionadas às condutas de manuseio da pessoa com COVID-19 também podem ter influenciado os achados. Sendo assim, tais dados não devem ser generalizados para centros hospitalares com características diferentes ou mesmo para a população mundial. Outra limitação refere-se ao uso de registros profissionais como fonte de dados, pois eles constituem uma ferramenta de trabalho e não de coleta rigorosa de informação para estudos. 

## Conclusão

O presente estudo confirmou a hipótese de que a infecção por COVID-19 não aumentou a chance de ocorrência de eventos tromboembólicos em pessoas com câncer. Para a população estudada, os fatores que tiveram associação com estes eventos foram os relacionados à doença neoplásica e aos respectivos tratamentos. Ademais, o estudo trouxe contribuição para a literatura, tendo em vista a lacuna em relação às pesquisas clínicas na área de enfermagem voltadas para essa população. Os achados do estudo podem nortear enfermeiros e toda equipe de saúde no planejamento do cuidado para alta complexidade identificada na população estudada, considerando a prevenção de eventos tromboembólicos. Sugere-se estudos clínicos que avaliem as medidas de prevenção desses eventos em população semelhante, a fim de identificar o impacto dessas medidas na ocorrência desses eventos.

# Translation: Eventos tromboembólicos en personas con cáncer durante la pandemia de COVID-19: estudio de caso-control*

**DOI:** 10.1590/1518-8345.7075.4265

**Authors:** Vivian Cristina Gama Souza Lima, Paulo Jorge Pereira Alves, Patrícia dos Santos Claro Fuly

**Affiliations:** 1 Universidade Federal Fluminense, Escola de Enfermagem Aurora de Afonso Costa, Niterói, RJ, Brasil.; 2 Ministério da Saúde, Instituto Nacional do Câncer, Rio de Janeiro, RJ, Brasil.; 3 Universidade Católica Portuguesa, Porto, Portugal.

**Keywords:** Enfermería Oncológica, Oncología, COVID-19, Trombosis, Neoplasias, Estudios de Casos y Controles

## Abstract

**Objetivo:**

analizar la asociación entre la infección por enfermedad por coronavirus y eventos tromboembólicos en personas con cáncer durante el primer año de la pandemia.

**Método:**

estudio caso-control realizado mediante la recolección de datos en historiales médicos. Los casos fueron adultos con cáncer, diagnosticados con evento tromboembólico, atendidos en las unidades del servicio seleccionado durante el primer año de la pandemia. El grupo control estuvo compuesto por adultos con cáncer sin diagnóstico de evento tromboembólico. Se aplicó la prueba de chi-cuadrado de Pearson para verificar la asociación entre factores de riesgo y el resultado, y se utilizaron técnicas de regresión logística para identificar la razón de posibilidades de ocurrencia de evento tromboembólico.

**Resultados:**

se incluyeron en el estudio 388 casos y 440 controles (proporción 1/1). Prevaleció el género femenino, raza blanca, con una edad media de 58,2 (±14,8) años. La quimioterapia antineoplásica fue el tratamiento más utilizado y la enfermedad por coronavirus fue identificada en el 11,59% de los participantes. En el grupo de casos, la trombosis venosa profunda fue más prevalente.

**Conclusión:**

el estudio confirmó la hipótesis de que la infección por enfermedad por coronavirus no aumentó la probabilidad de eventos tromboembólicos en personas con cáncer. Para la población estudiada, los factores que tuvieron asociación con estos eventos fueron los relacionados con el cáncer y su tratamiento.

## Destacados

**(1)** Trombosis venosa profunda prevaleció en la población estudiada.

**(2)** Quimioterapia aumentó la probabilidad de eventos tromboembólicos en un 65%.

**(3)** Eventos tromboembólicos mostraron una asociación significativa con la alta tasa de mortalidad.

**(4)** La covid-19 no aumentó el riesgo de evento tromboembólico en personas con cáncer.

## Introducción

El cáncer es uno de los principales problemas de salud pública en Brasil y en el mundo. Incluye más de 100 tipos de enfermedades y, junto con las enfermedades cardiovasculares, las enfermedades respiratorias y la Diabetes Mellitus forma parte del grupo de Enfermedades Crónicas No Transmisibles (ECNT), responsables de aproximadamente el 70% de todas las muertes en el mundo^1^.

La creciente incidencia de enfermedades oncológicas y la complejidad del cuidado a este individuo involucran varios aspectos, entre ellos el riesgo para el desarrollo de tromboembolismo venoso (TEV). Se sabe que existe una estrecha relación entre la enfermedad oncológica y el TEV, con un riesgo aumentado de que estas personas desarrollen un evento tromboembólico en comparación con la población en general. Un estudio de cohorte realizado en España comparó las tasas de incidencia de tromboembolismo en poblaciones con y sin cáncer entre los años 1997 y 2017. La incidencia acumulativa de TEV 12 meses después del diagnóstico de cáncer fue del 2,3% en la cohorte de cáncer y del 0,35% en la cohorte sin cáncer. Además, la incidencia de 12 meses en la cohorte de cáncer aumentó del 1,0% en 1997 al 3,4% en 2017, lo que sugiere que las nuevas terapias para el tratamiento del cáncer han alterado este riesgo^2^. El TEV es considerado la segunda causa más frecuente de muerte en personas con cáncer, además de ser responsable de mayores riesgos de complicaciones hemorrágicas durante el tratamiento anticoagulante y de trombosis venosa recurrente, que en personas sin neoplasia maligna^3^.

En este sentido, el cáncer y los diversos tratamientos son reconocidos como factores de riesgo independientes para el desarrollo de TEV. La asociación clínica entre cáncer e hipercoagulabilidad es conocida desde hace más de un siglo, y los eventos tromboembólicos son más frecuentes en personas con cáncer: una de cada cinco de ellas presentará TEV durante la evolución natural de la enfermedad^2^.

Existen varios mecanismos que se superponen e interactúan y pueden explicar el aumento de la incidencia de TEV en personas con cáncer. El cáncer, por sí solo, está asociado a un riesgo cuatro veces mayor de desarrollo del TEV, mientras que la quimioterapia antineoplásica aumenta este riesgo seis veces. Las personas en terapia con citotóxicos son responsables del 13% de los episodios de TEV en la población oncológica^2^.

Asociado a estos riesgos están las infecciones que afectan al individuo. En este contexto, recientemente, el mundo enfrentó una emergencia de salud pública con la pandemia de COVID-19 (enfermedad por coronavirus 2019). La COVID-19 es una enfermedad causada por el virus denominado SARS-CoV-2, y con alto potencial de contagio. La enfermedad surgió en 2019 en China y se esparció rápidamente por el planeta, causando síntomas respiratorios que pueden ser similares a un resfriado, gripe o neumonía^4^ ^)-(^ ^5^. La COVID-19 también se considera una enfermedad multisistémica, ocasionada, en gran parte, por la respuesta inmune del individuo y con involucramiento predominantemente endotelial^6^.

En cuanto al contexto del TEV, la COVID-19 es una enfermedad que puede provocar hiperinflamación y se ha asociado al riesgo aumentado de ocurrencia de fenómenos tromboembólicos, especialmente el tromboembolismo pulmonar, más frecuentemente observado en personas con neumonía grave, internadas en unidades de terapia intensiva^7^. Un estudio realizado en Francia, en el cual se realizaron 106 angiografías pulmonares en personas con COVID-19 durante el período de un mes, identificó 32 personas (30%) con embolia pulmonar aguda. Esta tasa de embolia pulmonar es mucho mayor que la generalmente encontrada en personas en cuidados críticos sin infección por COVID-19 (1,3%) o en personas en emergencia (3 a 10%). En cuanto a la trombosis arterial, se describen en personas con COVID-19, casos de accidente vascular cerebral isquémico agudo, debido a la obstrucción arterial de grandes vasos con incidencia mayor de lo habitual^6^.

Durante la pandemia de COVID-19, las personas con cáncer también fueron afectadas por esta nueva infección, sumando al cuadro de enfermedad crónica un riesgo adicional para la salud. Por lo tanto, se observa la importancia de identificar la especificidad de esta población, con el fin de orientar la asistencia a la persona con cáncer y el cuidado frente a la presencia de riesgos para el desarrollo de eventos tromboembólicos.

Al considerar los factores de riesgo de eventos tromboembólicos, reconocidos en personas con cáncer y los eventos tromboembólicos evidenciados en personas con COVID-19, este estudio pretende responder a la siguiente pregunta de investigación: ¿las personas con cáncer y diagnóstico de COVID-19 tienen un mayor riesgo de desarrollar eventos tromboembólicos? De esta forma, el objetivo de este estudio fue analizar la asociación entre la infección por COVID-19 y los eventos tromboembólicos en personas con cáncer, durante el primer año de la pandemia.

## Método

### Tipo de estudio

Estudio caso-control con proporción de 1 caso/1 control en personas adultas con cáncer. Esta proporción fue definida conforme al tiempo hábil para la realización de esta investigación. Se utilizaron las recomendaciones del *checklist Strengthening the Reporting of Observational Studies in Epidemiology* (STROBE)^8^ para la conducción y presentación del estudio.

### Lugar del estudio

El lugar de estudio fue un servicio de referencia en oncología de la red pública del estado de Río de Janeiro. Consiste en un complejo que reúne a través de cuatro unidades: 366 camas, incluyendo hospitalización clínica y terapia intensiva, red ambulatoria para consultas y exámenes de imagen y laboratorios, además de soporte de centro quirúrgico. La elección de este escenario se justifica por ser un servicio de salud de referencia en oncología, que también atendió a personas con cáncer que fueron afectadas por la COVID-19, durante la pandemia.

### Período

La recolección de datos se realizó entre abril de 2021 y diciembre de 2022.

### Población

Los casos se definieron como: adultos con cáncer, independientemente del diagnóstico oncológico, diagnosticados con cualquier tipo de evento tromboembólico registrado en prontuario. Se definieron controles como: adultos con cáncer, independientemente del diagnóstico oncológico, sin diagnóstico de evento tromboembólico. Se consideraron los siguientes criterios de inclusión: tener al menos 18 años y ser atendido en cualquier unidad del escenario de investigación en el primer año de la pandemia, comprendido entre el 11 de marzo de 2020, fecha inicial de la pandemia declarada por la Organización Mundial de la Salud (OMS)^9^, y el 11 de marzo de 2021, delimitando así el primer año de la pandemia. Este recorte temporal se justifica por los cambios que ocurrieron en el segundo año de la pandemia como el inicio de la vacunación y el cambio en el método diagnóstico con la prueba rápida, los cuales podrían interferir en los hallazgos. Se excluyeron los prontuarios que no fueron localizados o no presentaron información capaz de proporcionar los datos necesarios para el estudio, como los incompletos, inconclusos o ilegibles.

### Definición de la muestra

Para identificar la muestra de este estudio, se realizó una búsqueda previa en la base electrónica de prontuarios de la institución por términos que sugirieran los eventos tromboembólicos en el período propuesto para la recolección. De esta forma, se encontraron 7.297 ocurrencias. Al excluir las duplicaciones -personas con más de una ocurrencia- se llegó al total de 2.986 prontuarios que poseían estos términos. Se procedió a la lectura de las ocurrencias y, del total, 455 prontuarios contenían informes concluyentes de eventos tromboembólicos. De esta forma se definió 455 prontuarios también en el grupo control, totalizando 910 prontuarios. Tras la aplicación de los criterios de exclusión, la muestra de interés con evento tromboembólico estuvo compuesta por 388 prontuarios (grupo caso) y 440 para el grupo control, totalizando 828 prontuarios incluidos en la investigación.

El emparejamiento de las muestras entre los grupos caso y control buscó similitud entre los individuos, de forma que los controles fueran similares a los casos en relación a ciertas características que no aquella que trata del factor bajo investigación. Así, la definición del grupo control en relación al grupo caso en este estudio ocurrió por la comparación entre las variables sexo y edad, mostrando, inicialmente, que ambos grupos eran de población similar, en lo que se refiere a las distribuciones de estas variables. Para esto, conforme los prontuarios seleccionados se insertaban en los grupos determinados, se extraían informes parciales, a fin de verificar la homogeneidad de los grupos en relación con estas variables. Al observar diferencia entre los grupos, los participantes eran excluidos o incluidos, a fin de equilibrar numéricamente los mismos, conforme a las variables citadas (Figura 1).

**Figura 1:** Selección de los prontuarios para el estudio. Río de Janeiro, RJ, Brasil, 2023

### Variables del estudio

En este estudio, se consideró el diagnóstico de cualquier evento tromboembólico - incluyendo arteriales y venosos, como variable de resultado y el diagnóstico de COVID-19 como variable indicadora para la determinación de los subgrupos. Las demás variables investigadas fueron definidas con el objetivo de caracterizar a las personas, de acuerdo con el perfil sociodemográfico y clínico, además de identificar los factores de riesgo para eventos tromboembólicos.

Se seleccionaron variables orientadas a la caracterización de la clientela: edad, estado civil, género, raza/color, peso, altura, *Performance Status* (PS); y características clínicas y factores de riesgo para TEV: comorbilidades, cáncer activo (sí o no), neoplasia maligna (sí o no), sitio tumoral cerebro, páncreas, estómago, pulmón, vejiga, ginecológicos, hematológicos y “otros”), tipo de tratamiento (cirugía, quimioterapia antineoplásica, radioterapia, etc), uso de tabaco, inmovilidad, resultados de exámenes laboratoriales, historial de TEV, traumas recientes, uso de medicamentos (anticonceptivo oral; agentes estimulantes de la eritropoyesis), factores genéticos - mutaciones de los genes Factor II - Protrombina, Factor V de Leiden^10^ u otros- y puntuación según la escala de Padua.

La Escala de *Performance Status del Eastern Cooperative Oncology Group* (PS-ECOG) evalúa cómo la enfermedad afecta las habilidades de vida diaria de la persona, con una puntuación que varía de cero a cinco puntos^11^. Se optó por esta variable, ya que es utilizada en la institución escenario del estudio registrada en prontuario, idealmente hecha en cada evaluación clínica. La Escala de Padua es una escala sugerida por el *American College of Chest Physicians* (ACCP), como forma de evaluación de riesgo para TEV. Esta escala evalúa los 14 factores de riesgo, en que cada factor puntuado se suma para generar un riesgo acumulativo. La puntuación final define el nivel de riesgo de TEV del individuo, siendo una puntuación ≥ 4 alto riesgo y una puntuación < 4 bajo riesgo^12^. Estas escalas no fueron validadas específicamente para la población de este estudio y fueron utilizadas como variables por tener sus datos disponibles en prontuario y su medición no sugiere riesgo al participante de la investigación.

### Instrumentos utilizados para la recolección de la información

Los datos se recogieron a partir de fuentes secundarias, siendo estos los prontuarios de las personas atendidas en el servicio en el intervalo de tiempo definido para esta investigación. Se utilizó un formulario informatizado en línea, alojado en un sitio de acceso exclusivo del equipo de investigación creado específicamente para este estudio, que recoge los datos sociodemográficos y clínicos, elaborado a partir de las variables descritas anteriormente conforme a la literatura.

### Recolección de datos

La recolección contó con cuatro asistentes de investigación, participantes como becarios de iniciación científica en el proyecto de esta investigación, previamente entrenados *in situ* por la investigadora principal para la inserción de los datos, los cuales quedaron almacenados en la base de datos gestionada por la investigadora principal. Los asistentes de investigación se distribuyeron entre la recolección y doble chequeo de los datos para garantizar la veracidad de los hallazgos. Fueron cegados para los objetivos de este estudio, así como para la pregunta de investigación, como forma de minimizar el sesgo de confusión, en el intento de realizar alguna asociación entre las respuestas. La elección de la recolección en prontuarios consideró minimizar el sesgo de memoria, que a veces ocurre cuando una persona intenta recordar los hechos ocurridos durante la hospitalización. El uso de registros profesionales como fuente de datos se configura como posible sesgo, ya que estos constituyen herramienta de trabajo y no de recolección rigurosa de información para estudios. En este caso, la ausencia o el error de registro de algunas informaciones pueden influir en los hallazgos. 

### Tratamiento y análisis de los datos

La versión final de la base de datos fue transportada del *Microsoft Excel* ^*®*^ al *software Stata* versión 16.0. En el análisis descriptivo, se procedió a la presentación de la distribución de las informaciones sociodemográficas, nutricionales, clínicas, de hábitos de vida, de tratamiento y ocurrencia de eventos. Para las variables cualitativas (diagnóstico y tipo de evento tromboembólico, diagnóstico de COVID-19, comorbilidades, neoplasia maligna, cáncer activo, sitio tumoral, tratamiento, quimioterapia, cirugía o trauma reciente, TEV previo, tabaquismo, uso de medicamentos, inmovilización prolongada, uso de catéter venoso central, factores genéticos y otros), se calcularon los números absolutos y las frecuencias. Para las variables cuantitativas (edad, peso, altura, PS, plaquetas, hemoglobina, d-dímero y Escala de Padua), se calcularon medidas de posición y dispersión. Se aplicó la prueba de independencia, tipo chi-cuadrado de Pearson, para verificar la presencia de asociación entre factores de riesgo, considerados variables independientes, y el desenlace de interés, adoptado en este estudio, como la ocurrencia de evento tromboembólico de cualquier tipo. Se aplicaron técnicas de regresión logística para identificar la razón de probabilidades de ocurrencia de evento tromboembólico en estratos muestrales de interés, siendo considerada como categoría de referencia el “No” o la categoría que representa, según la literatura, el menor riesgo o el mejor desenlace.

El nivel de significancia adoptado en todo el análisis fue del 5% y se utilizaron tablas para presentar los resultados. Al observar datos ausentes en algunos participantes, se optó por una prueba. La base de datos fue probada para las variables con datos ausentes, considerando la estructura original con todos los participantes y una composición alternativa, en la cual se retiraron los participantes con datos ausentes. La prueba chi-cuadrado mostró que los patrones de asociación entre las variables independientes y la variable dependiente no cambiaron, lo que permitió inferir la baja repercusión de los datos ausentes en el aspecto de las asociaciones.

### Aspectos éticos

Se presentaron todas las declaraciones y términos de responsabilidad del investigador conforme exigencia de la institución escenario del estudio. El estudio fue aprobado por los Comités de Ética en Investigación de las instituciones involucradas, conforme los dictámenes números 4.486.636 y 4.509.083. 

## Resultados

Se incluyeron un total de 828 prontuarios en el estudio. Los participantes fueron mayoritariamente del género femenino (65%) y de raza blanca (68%). La edad media de los participantes fue de 58,2 (±14,8) años. La comorbilidad más prevalente entre los participantes fue la hipertensión, presente en el 43,5%, seguida de la diabetes (18%). Otras comorbilidades estuvieron presentes en el 39,4% de los participantes. El diagnóstico de COVID-19 fue confirmado en el 11,59% de los participantes en general y la tasa de mortalidad global fue del 35,51% (Tabla 1).

**Tabla 1:** Caracterización sociodemográfica y clínica de los participantes del estudio (n = 828). Rio de Janeiro, RJ, Brasil, 2021-2022

Variables			n	%
Sexo (n=828)				
Femenino			539	65,10
Masculino			289	34,90
Raza (n=828)				
Blanca			564	68,12
Parda			204	24,64
Negra			58	7,0
Edad superior a 70 años (n=826)				
Sí			144	17.43
No			682	82.57
Tabaquismo (n=828)				
Sí			148	17.87
No			680	82.13
Clasificación de IMC (n=760)				
Bajo peso severo			12	1.58
Bajo peso moderado			14	1.84
Bajo peso leve			36	4.74
Adecuado			307	40.39
Sobrepeso			244	32.11
Obesidad grado 1			127	16.71
Obesidad grado 2			35	4.60
Obesidad grado 3			12	1.57
Obesidad (n=760)				
Sí			174	22,86
No			587	77,14
COVID-19				
Sí			96	11,59
No			732	88,41
Óbito				
Sí			294	35,51
No			534	64,49
Hipertensión Arterial				
Sí			360	43,48
No			468	56,52
Diabetes Mellitus				
Sí			149	18,0
No			679	82,0
*Performance status*				
0			100	12,08
1			350	42,27
2			132	15,94
3			117	14,13
4			129	15,58
**Variable**	Promedio (±DE*)	**Mediana (IIQ** ^†^ **)**	**Mínimo**	**Máximo**
Edad	58,22 (±14,8)	60 (72)	18	91

*Desviación-estándar; ^†^Rango intercuartil

Del total de participantes, el 90,5% tenían neoplasia maligna, el 73,91% tenía cáncer activo y el sitio tumoral más frecuente fue el ginecológico (17,1%), seguido por el de mama (16,06%). La metástasis estaba presente en el 24,6% de la muestra. De las 828 personas, 135 tenían resultados de exámenes laboratoriales con valores de D-dímero. Se notaron elevadas tasas de este marcador en la población estudiada, siendo la media de 5.006,66 ng/ml, alcanzando valores de hasta 33.441 ng/ml. Los valores medios de hemoglobina fueron de 11,71 g/dL (n=815) y de plaquetas pre-quimioterapia antineoplásica de 281,09 mil/mm³ (n=541). La quimioterapia antineoplásica fue el tratamiento más frecuentemente adoptado (68,2%), seguida por la cirugía (43,7%) y la radioterapia (40,82%). Quimioterapias antineoplásicas recientes fueron realizadas en el 26,9% de los participantes (Tabla 2).

**Tabla 2:** Caracterización clínica de la enfermedad oncológica y tratamientos de las personas con cáncer (n = 810). Rio de Janeiro, RJ, Brasil, 2021-2022

Variables			n	%
Neoplasia maligna				
Sí			749	90,46
No			79	9,54
Cáncer activo				
Sí			612	73,91
No			216	26,09
Sitio tumoral: ginecológico				
Sí			142	17,15
No			686	82,85
Sitio tumoral: hematológico				
Sí			70	8,45
No			758	91,55
Sitio tumoral: mama				
Sí			133	16,06
No			695	83,94
Metástasis				
Sí			204	24,64
No			624	75,36
Quimioterapia antineoplásica				
Sí			565	68,24
No			263	31,76
Radioterapia				
Sí			338	40,82
No			490	59,18
Cirurgía				
Sí			362	43,72
No			466	56,28
Uso del catéter venoso central				
Sí			104	12,56
No			724	87,44
**Variables**	Promedio (±DE*)	**Mediana (IIQ** ^†^)	**Mínimo**	**Máximo**
Plaquetas pre-quimioterapia antineoplásica (N=541)	281,09 (±313,88)	245 (824)	4	533,30
Hemoglobina (N=815)	11,71 (±6,91)	11,6 (81,0)	3,1	12,7
D-dímero (N=135)	5.006,66 (±6.240,76)	2.988 (22.490)	155	33.441
Tiempo de hospitalización (N=326)	12,68 (±15,86)	7,5 (74)	1	160
Escala de Pádua (N=810)	3,43 (2,18)	3 (10)	1	11

*Desviación-estándar; ^†^Rango intercuartil

En relación con otros procedimientos terapéuticos también considerados factores de riesgo para eventos tromboembólicos, el 13,41% de los participantes realizó hemotransfusiones.

### Comparación entre los grupos caso y control

Al considerar solo el grupo caso, el evento más incidente en la población estudiada fue la trombosis venosa profunda (65,98%), seguido de la embolia pulmonar (EP) (6,96%) y el accidente vascular cerebral (AVC) (3,61%). Los eventos de coagulación intravascular diseminada e infarto agudo de miocardio (IAM) estuvieron presentes en menor frecuencia, 0,26% (n=1) y 1,8% (n=7) respectivamente. Otros eventos sumaron el 21,39%.

En relación con las características clínicas de las neoplasias, se identificó que la presencia de neoplasia maligna y de cáncer activo estaban en mayor ocurrencia en el grupo caso, lo que infiere que están asociados al evento tromboembólico. La probabilidad de ocurrencia de este evento en individuos con neoplasia maligna es un 79% mayor en comparación con aquellos que no la poseen, mientras que en personas con cáncer activo la probabilidad de presentación del evento tromboembólico es tres veces mayor en comparación con quienes no poseen cáncer activo. En cuanto a la escala de riesgo de Padua, no hubo asociación significativa con el evento en la población estudiada (Tabla 3).

**Tabla 3:** Asociación entre características clínicas y ocurrencia de evento tromboembólico de cualquier tipo (n = 828). Río de Janeiro, RJ, Brasil, 2021-2022

Variables	Grupo				OR*	IC95%^†^	p^‡^
	Caso		Control				
	n	%	n	%			
Neoplasia maligna							
Sí	361	48,20	388	51,8	1,791	1,101-2,914	0,018
No	27	34,18	52	65,82	1	-	
Cáncer activo							
Sí	334	54,58	278	45,42	3,604	2,548-5,097	<0,001
No	54	25,0	162	75,0	1	-	
No	368	46,0	432	54,0	1	-	
Sitio tumoral: ginecológico							
Sí	78	54,93	64	45,07	1,478	1,028-2,125	0,034
No	310	45,19	376	54,81	1	-	
Sitio tumoral: hematológico							
Sí	19	27,14	51	72,86	0,392	0,227-0,677	0,001
No	369	48,68	389	51,32	1	-	
Sitio tumoral: mama							
Sí	42	31,58	91	68,42	0,465	0,313-0,690	<0,001
No	346	49,78	349	50,22	1	-	
Escala de Pádua (n=723)							
Hasta 3	185	49,47	189	50,53	1	-	0,571
Igual o mayor a 4	180	51,58	169	48,42	0,919	0,686; 1,23	

* *Odds ratio* (Razón de probabilidades); ^†^Intervalo de confianza de 95%; ^‡^Prueba chi-cuadrado

En cuanto a los tratamientos realizados en personas con cáncer, se identificó, en el grupo caso, en relación con el control, que la realización de quimioterapia antineoplásica, quimioterapia antineoplásica reciente, transfusiones sanguíneas y terapia endocrina están asociadas a la ocurrencia de evento tromboembólico. La probabilidad de ocurrencia de estos eventos en personas que realizan quimioterapia antineoplásica fue un 65% mayor, en comparación con aquellos que no la realizan, mientras que en personas en quimioterapia antineoplásica reciente, la probabilidad de presentación del evento tromboembólico fue un 78% superior en comparación con quienes no la realizaban (Tabla 4).

**Tabla 4:** Asociación entre características de tratamiento y ocurrencia de evento tromboembólico de cualquier tipo (N = 828). Río de Janeiro, RJ, Brasil, 2021-2022

Variables	Grupo				OR*	IC95%^†^	p^‡^
	Caso		Control				
	n	%	n	%			
Quimioterapia antineoplásica							
Sí	287	50,8	278	49,2	1,655	1,228-2,231	0,001
No	101	38,4	162	61,6	1	-	
Terapia endócrina							
Sí	35	36,46	61	63,54	0,616	0,396-0,956	0,030
No	353	48,22	379	51,78	1	-	
Quimioterapia antineoplásica reciente							
Sí	128	57,40	95	42,6	1,787	1,310-2,438	<0,001
No	260	42,98	345	57,02	1	-	
Transfusiones							
Sí	63	56,76	48	43,24	1,583	1,057-2,369	0,025
No	325	45,33	392	54,67	1	-	
Hemoglobina (n=816)							
< 10	132	57,39	98	42,61	1,772	1,302-2,412	<0,001
≥ 10	253	43,17	333	56,83	1	-	
COVID-19							
Sí	41	42,71	55	57,29	0,827	0,538-1,270	0,386
No	347	47,4	385	52,6	1	-	
Uso del Catéter Venoso Central							
Sí	49	47,12	55	52,88	1,011	0,670; 1,527	0,955
No	339	46,82	385	53,18	1	-	
Desenlace de óbito							
Sí	180	61,22	326	61,05	2,474	1,847-3,314	<0,001
No	280	38,95	114	38,78	1	-	
Performance status							
0	36	36,0	64	64,0	1	-	<0,001
1	143	40,86	207	59,14	1,228	0,774-1,946	
2	58	43,94	74	56,06	1,393	0,817-2,376	
3	73	62,39	44	37,61	2,949	1,695-5,131	
4	78	60,47	51	39,53	2,718	1,584-4,664	

*Odds ratio (Razón de probabilidades); ^†^Intervalo de confianza de 95%; ^‡^Prueba chi-cuadrado

En cuanto a la COVID-19, el 42,71% de las personas con diagnóstico confirmado estaban en el grupo caso, sin embargo, la asociación entre la COVID-19 y el evento tromboembólico no mostró significancia estadística. De las personas que fallecieron, el 61,22% formaba parte del grupo caso y esto mostró relevancia desde el punto de vista estadístico entre estas dos condiciones. De igual manera, de las personas en seguimiento ambulatorio, el 38% formaba parte del grupo caso y las personas con valores de performance status 3 y 4 estaban más presentes en el grupo caso, mostrando una relación estadísticamente significativa de este indicador con la ocurrencia de estos eventos (Tabla 4).

La ocurrencia de cualquier tipo de evento tromboembólico (ET) tuvo una asociación significativa con la mortalidad, aunque no fue el objetivo de este estudio determinar qué evento específico tuvo una mayor correlación con este resultado. De las personas que experimentaron un ET, el 46,39% falleció. Además, la hemoglobina tuvo una asociación estadística significativa con el evento. Las personas con hemoglobina menor que 10 tuvieron una mayor probabilidad de desarrollar eventos tromboembólicos. La condición de la hemoglobina (menor o mayor que 10) está asociada con la ocurrencia de un evento tromboembólico. La razón de posibilidades para la ocurrencia de ET fue un 77% mayor en personas con hemoglobina menor que 10 (IC95%: 1,302-2,412; p<0,001), de acuerdo con la Tabla 4.

## Discusión

El estudio explora la ocurrencia de eventos tromboembólicos y las características clínicas de personas con cáncer en el contexto de la pandemia de COVID-19 en Brasil y, al caracterizar los aspectos clínicos de personas portadoras de neoplasias malignas según la ocurrencia o no de estos eventos, considerando las variables sexo y edad, se observó que los grupos caso y control fueron homogéneos entre sí. Además, las tasas de fallecimientos no fueron diferentes entre los grupos, reforzando la complejidad de la enfermedad oncológica. Como hallazgo en el estudio actual, se tienen diversos tipos de tratamiento para el cáncer como factores asociados a una mayor probabilidad de ocurrencia de eventos tromboembólicos.

En el contexto de personas con cáncer también portadoras de otras comorbilidades, se percibe el potencial para aumento del riesgo de complicaciones en la población estudiada. En este aspecto, se observa en la literatura un estudio que utilizó una medida compuesta para evaluar la necesidad de cuidados intensivos (unidad de cuidados intensivos - UCI y ventilación mecánica) y fallecimiento como medida de riesgo de gravedad de la infección por COVID-19, e identificó un riesgo 79% mayor en individuos con cualquier comorbilidad, incluyendo cáncer, diabetes, hipertensión, enfermedades cardiovasculares y cerebrovasculares, en comparación con individuos sin comorbilidades. Para personas con dos o más comorbilidades asociadas, el riesgo fue 2.5 veces más elevado^13^.

En cuanto a la incidencia de los tipos de cáncer con mayor riesgo para el desarrollo de eventos tromboembólicos, se observó, en esta investigación, una mayor frecuencia de tumores ginecológicos (17,15%), mama (16,06%) y hematológico (8,45%), respectivamente. En este sentido, se identificó en la literatura un estudio prospectivo y observacional de 10,684 pacientes con trombosis. En este estudio, 1,075 pacientes poseían cáncer activo, y entre los sitios más prevalentes se observó mama (10,6%) y tumores ginecológicos (10,3%)^14^. El cáncer de mama también estuvo entre los más incidentes en un estudio realizado en California con una tasa del 12% entre los que tuvieron eventos tromboembólicos^15^. No obstante, se destaca que la incidencia reportada de trombosis venosa asociada al cáncer varía ampliamente entre los diversos estudios debido a diferencias en la determinación y en las poblaciones subyacentes representadas.

La probabilidad de ocurrencia de un evento tromboembólico en individuos con neoplasia maligna en este estudio fue un 79% mayor en comparación con aquellos que no la poseen, según los resultados de este estudio. Además, las personas con cáncer activo presentaron tres veces más probabilidad de evento tromboembólico en comparación con quienes no tienen cáncer activo. Este dato puede ser confirmado en la literatura, ya que está asociado con los factores biológicos que causan el riesgo de trombosis en personas con cáncer. Estos factores incluyen la activación de la trombina y la formación de fibrina. Esta activación se produce directamente por la liberación de factores procoagulantes y citoquinas que son producidas por las células tumorales. Las citoquinas estimulan a las células endoteliales íntegras y los monocitos para expresar el factor tisular en la membrana externa, lo que resulta en la activación de la cascada de coagulación^16^.

En cuanto a los eventos tromboembólicos y los valores de hemoglobina, los hallazgos de esta investigación corroboran la literatura, que observa que las personas con cáncer tienen un riesgo sustancialmente más elevado para episodios nuevos y recurrentes de trombosis venosa profunda, en comparación con personas sin cáncer^3^. Además, las personas con hemoglobina menor de 10 tuvieron una mayor probabilidad de desarrollar eventos tromboembólicos. Este dato confirma este indicador como un factor de riesgo para eventos tromboembólicos, según el estudio de validación de la Escala de Predicción de Riesgo de Khorana^17^.

La trombosis venosa profunda fue el evento más prevalente en la población estudiada (65,98%), seguida de la embolia pulmonar (6,96%). De acuerdo con la literatura, las tasas de mortalidad por trombosis venosa profunda atribuidas a causas idiopáticas son más bajas que las observadas en personas con cáncer. Hasta un 20% de las personas con cáncer desarrollarán eventos tromboembólicos y el riesgo de trombosis aumenta en pocos meses (de cero a tres meses) después del diagnóstico de malignidad y con la presencia de metástasis^16^. Es destacable, en el estudio en cuestión, que los eventos tromboembólicos estuvieron asociados con el fallecimiento, siendo más relevantes que la infección por COVID-19 en la población estudiada.

La incidencia de uso de catéteres venosos centrales en la población de este estudio fue baja y, en consecuencia, los eventos tromboembólicos relacionados con catéteres no estuvieron tan presentes en esta población. Este dato se correlaciona con el contexto de la pandemia, en el cual el servicio de salud escenario del estudio priorizó atenciones quirúrgicas de urgencia, impactando en la inserción de catéteres. El manejo de catéteres venosos centrales es un elemento fundamental en el tratamiento del paciente con cáncer por ser una vía importante para su tratamiento. La trombosis asociada a catéter es frecuentemente observada en pacientes con enfermedades malignas; sin embargo, a pesar de ser una complicación común entre estos pacientes, la información objetiva sobre su epidemiología, evolución clínica, profilaxis y estrategias de tratamiento aún es muy limitada en la literatura actual^18^.

La población de esta investigación mostró resultados de exámenes con valores altos de D-dímero. En el contexto de COVID-19, se produce un estado de hipercoagulabilidad y alteraciones hematológicas, que han sido descritas en hasta un tercio de las personas, siendo la elevación de los niveles de D-dímero un importante marcador de desenlaces desfavorables^19^. Ante esto, algunas series retrospectivas investigaron la frecuencia de tromboembolismo venoso en personas con COVID-19, identificando la presencia de alteraciones en hasta el 40% de las personas^20^.

En este estudio, la elevación del marcador D-dímero no demostró ser relevante en la ocurrencia de eventos tromboembólicos. Esta condición es común en COVID-19, lo cual dificulta su uso en la investigación de otros eventos, como la trombosis, por ejemplo^19^. La tasa de mortalidad global fue alta (35,51%), considerando el contexto de COVID-19. Un estudio con personas en tratamiento clínico mostró una tasa del 12%, la más elevada en personas mayores en el escenario de la pandemia^21^. Sin embargo, la tasa de mortalidad de este estudio se aproxima más a las tasas de mortalidad evidenciadas en personas con cáncer, independientemente de la infección por COVID-19^22^.

La realización de quimioterapia antineoplásica, quimioterapia antineoplásica reciente, transfusiones sanguíneas y terapia endocrina mostraron una asociación significativa con la ocurrencia de eventos tromboembólicos. Se observó que en este estudio, la probabilidad de ocurrencia de estos eventos en personas que realizaron quimioterapia antineoplásica fue un 65% mayor en comparación con aquellos que no la realizaron, mientras que en personas en quimioterapia antineoplásica reciente, la probabilidad de presentación del evento tromboembólico fue un 78% superior en comparación con quienes no la realizaron.

En relación con el tratamiento del cáncer, diversos factores contribuyen al evento tromboembólico. Las anomalías de las paredes de los vasos son consecuencias de los daños de la enfermedad oncológica, ya sea por la quimioterapia antineoplásica, por las cirugías o por el uso de catéter venoso. Además, los agentes quimioterapéuticos también están asociados con la hipercoagulación, por la reducción de niveles plasmáticos de anticoagulantes fisiológicos y por el efecto citotóxico de la quimioterapia antineoplásica que aumenta la expresión del factor tisular y la actividad procoagulante^16^.

En este contexto, este resultado es corroborado por una cohorte reciente, el *COVID-19 and Cancer Consortium* (CCC19), que incluyó a 1.629 personas con cáncer hospitalizadas con COVID-19 y concluyó que la terapia anticancerígena reciente, el cáncer activo, los subtipos de cáncer con alto riesgo para TEV y la hospitalización en UCI estuvieron asociados con el riesgo aumentado de TEV y EP. En contraste, en este estudio, se evidenció que la terapia anticoagulante o antiplaquetaria previa a la admisión podría reducir ese riesgo^23^.

Se observa, de esta manera, que el cáncer y los respectivos tratamientos mostraron mayor relevancia frente a la infección por COVID-19 en relación con los eventos tromboembólicos. En este sentido, la evaluación de los factores de riesgo para estos eventos a través de herramientas estratégicas sigue siendo una recomendación importante. Para ello, escalas de evaluación de riesgo han sido validadas para personas sometidas a procedimientos quirúrgicos, personas en curso de tratamiento antineoplásico, personas hospitalizadas, entre otras condiciones clínicas a considerar^24^ ^)-(^ ^25^. Todas estas formas de evaluación incluyen el cáncer como uno de los factores de riesgo y corresponde al profesional elegir la que más se adecue a la persona bajo su cuidado. Cabe destacar que en este estudio, la escala de Padua no mostró asociación estadística significativa en la ocurrencia de eventos tromboembólicos, lo que infiere que para esta población, lo ideal sería usar otra escala ya validada. Actualmente, solo la escala de riesgo de Khorana está validada para personas con cáncer, pero solo a nivel ambulatorio, sin considerar aspectos relacionados con la hospitalización y cirugías, por ejemplo^17^.

Además de las evaluaciones de riesgo, se pueden trazar otras estrategias preventivas para las personas con cáncer y consecuente riesgo de evento tromboembólico. El uso de medias de compresión, el estímulo a la deambulación precoz, la movilización en la cama y el análisis de exámenes de laboratorio deben emplearse en la rutina del profesional de salud, sea médico o enfermero, con el fin de identificar riesgos y señales de alerta en esta población^26^. Otra acción del equipo multidisciplinario es la orientación a este individuo y su familia, ya que la quimioterapia antineoplásica, sea reciente o no, fue un factor que aumentó la probabilidad de ocurrencia del evento tromboembólico y, considerando que una parte considerable de las personas con cáncer en tratamiento antineoplásico lo hacen ambulatoriamente, es decir, tienen este riesgo en sus residencias, corresponde al equipo de salud orientar sobre los signos de alerta que deben ser considerados para la búsqueda inmediata del servicio de salud^26^.

De esta forma, al considerar los descubrimientos sobre la COVID-19 en cuanto a los mecanismos de transmisibilidad, la fisiopatología de la enfermedad, los tratamientos y las medidas de prevención, protección y control, la experiencia de la pandemia reveló nuevos hallazgos. El enfrentamiento de la pandemia de COVID-19 requirió dinamismo y reestructuración de los servicios para responder a las necesidades de la población, evidenciándose nuevos arreglos tecnoasistenciales tanto en el ámbito de la gestión como de la atención a la salud, siendo este un gran desafío en pro de la promoción de la salud de las personas^27^. Por ese motivo, identificar la característica de estas personas se vuelve tan relevante. Algunos signos y síntomas clásicos de la COVID-19 pueden ser también síntomas de complicaciones del propio tratamiento oncológico o del padecimiento por cáncer, exigiendo del profesional la elaboración de una evaluación de riesgo basada en evidencias, para que se implementen las acciones específicas, con foco en la necesidad del individuo en ese momento^19^.

Las limitaciones del estudio están relacionadas con la metodología de un estudio observacional y retrospectivo. El período y la población restringida pueden influir directamente en los resultados y en los intervalos de confianza. Además, las diversas adaptaciones de los servicios de salud implementadas en el primer año de la pandemia, relacionadas con las conductas de manejo de la persona con COVID-19 también pueden haber influido en los hallazgos. Por lo tanto, estos no deben generalizarse para centros hospitalarios con características diferentes y para la población mundial. Otra limitación se refiere al uso de registros profesionales como fuente de datos, ya que constituyen una herramienta de trabajo y no de recolección rigurosa de información para estudios.

## Conclusión

El presente estudio confirmó la hipótesis de que la infección por COVID-19 no aumentó la probabilidad de ocurrencia de eventos tromboembólicos en personas con cáncer. Para la población estudiada, los factores que tuvieron asociación con estos eventos fueron los relacionados con la enfermedad neoplásica y los respectivos tratamientos. Además, el estudio aportó una contribución a la literatura, teniendo en cuenta la brecha en relación con las investigaciones clínicas en el área de enfermería dirigidas a esta población. Los hallazgos del estudio pueden guiar a enfermeros y a todo el equipo de salud en la planificación del cuidado para la alta complejidad identificada en la población estudiada, teniendo en cuenta la prevención de eventos tromboembólicos. Se sugieren estudios clínicos que evalúen las medidas de prevención de estos eventos en una población similar, con el fin de identificar el impacto de estas medidas en la ocurrencia de estos eventos.
